# Structure-Based Virtual Screening for Methyltransferase Inhibitors of SARS-CoV-2 nsp14 and nsp16

**DOI:** 10.3390/molecules29102312

**Published:** 2024-05-15

**Authors:** Kejue Wu, Yinfeng Guo, Tiefeng Xu, Weifeng Huang, Deyin Guo, Liu Cao, Jinping Lei

**Affiliations:** 1 Guangdong Key Laboratory of Chiral Molecule and Drug Discovery, School of Pharmaceutical Sciences, Sun Yat-Sen University, Guangzhou 510006, China; wukj6@mail2.sysu.edu.cn (K.W.); guoyf36@163.com (Y.G.); huangwf26@mail2.sysu.edu.cn (W.H.); 2 Centre for Infection and Immunity Studies (CIIS), School of Medicine, Sun Yat-Sen University, Shenzhen 518107, Chinaguo_deyin@gzlab.ac.cn (D.G.); caoliu@mail.sysu.edu.cn (L.C.); 3 Guangzhou Laboratory, Bio-Island, Guangzhou 510320, China

**Keywords:** SARS-CoV-2, nsp14, nsp16, MTase inhibitors, structure-based virtual screening

## Abstract

The ongoing COVID-19 pandemic still threatens human health around the world. The methyltransferases (MTases) of SARS-CoV-2, specifically nsp14 and nsp16, play crucial roles in the methylation of the N7 and 2′-O positions of viral RNA, making them promising targets for the development of antiviral drugs. In this work, we performed structure-based virtual screening for nsp14 and nsp16 using the screening workflow (HTVS, SP, XP) of Schrödinger 2019 software, and we carried out biochemical assays and molecular dynamics simulation for the identification of potential MTase inhibitors. For nsp14, we screened 239,000 molecules, leading to the identification of three hits A1–A3 showing N7-MTase inhibition rates greater than 60% under a concentration of 50 µM. For the SAM binding and nsp10-16 interface sites of nsp16, the screening of 210,000 and 237,000 molecules, respectively, from ZINC15 led to the discovery of three hit compounds B1–B3 exhibiting more than 45% of 2′-O-MTase inhibition under 50 µM. These six compounds with moderate MTase inhibitory activities could be used as novel candidates for the further development of anti-SARS-CoV-2 drugs.

## 1. Introduction

According to the latest statistics of the COVID-19 pandemic (https://covid19.who.int/, accessed on 25 February 2024), there are more than 774 million people globally suffering from infections of severe acute respiratory syndrome coronavirus 2 (SARS-CoV-2), which has caused about 7.01 million deaths as of 25 February 2024 [1]. Compared to the earlier Middle East respiratory syndrome coronavirus (MERS-CoV) and severe acute respiratory syndrome coronavirus 1 (SARS-CoV-1) pandemics, the current COVID-19 pandemic poses a substantial threat to human health [2,3]. As SARS-CoV-2 continues to spread and undergo mutations to a variety of variants, such as Delta and Omicron, the demand for effective vaccines as well as antiviral therapeutic drugs increases drastically [4,5]. However, there is no specific drug to cure SARS-CoV-2 infection currently, and only a few antiviral agents such as Remdesivir, Azvudine, Paxlovid, and Molnupiravir have been approved for the treatment of COVID-19 in adult and pediatric patients [6,7,8,9].

**Figure 1:**
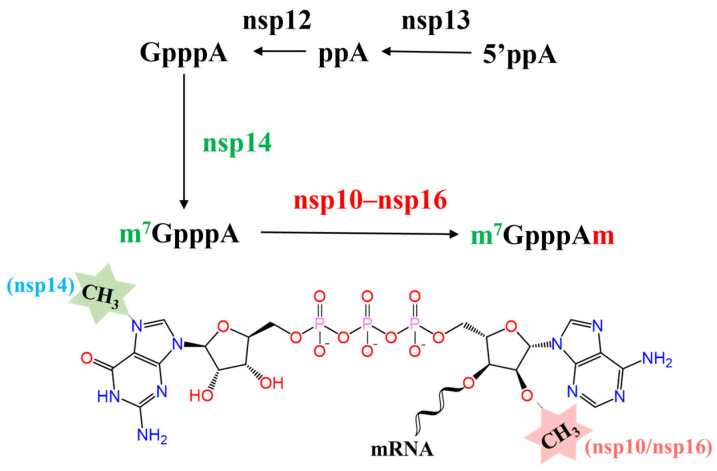
SARS-CoV-2 capping outline. The initial cap core structure (cap-0) of SARS-CoV-2 is formed at the 5′-end of RNA. First, the newly generated RNA is hydrolyzed into ppRNA by RNA 5′-triphosphatase (RTPase/nsp13), and the terminal γ-phosphate is removed. Then, Gppp-RNA is formed under the catalysis of guanylyltransferase (GTase/nsp12). Subsequently, the N7-position is methylated by N7-MTase (nsp14) to form the cap-0 structure. Finally, nsp10–16 catalyzes the formation of the ultimate cap-1 structure.

SARS-CoV-2 is a positive-strain RNA virus with a genomic size of approximately 29.9 kb that is extremely contagious [10]. The first two-thirds of the SARS-CoV-2 genome is composed of a pair of large open reading frames (Orf1a and Orf1ab), whose encoded polyproteins (pp1a and pp1ab) are decomposed into 16 different non-structural proteins (nsp1 to nsp16) that are responsible for viral replication and transcription in eukaryotic cells [11]. Among these nsp proteins, nsp12 (RNA guanylyltransferase), nsp13 (RNA triphosphatase), nsp14 (RNA guanine-N7-methyltransferase, N7-MTase), and nsp16 (RNA 2′-O-methyltransferase, 2′-O-MTase) are involved in viral mRNA capping (Figure 1), which helps the SARS-CoV-2 virus escape the administration of the host innate immune system.

nsp14 is an N7-MTase that converts SARS-CoV-2 viral mRNA to a cap-0 structure, while the 2′-O-MTase nsp16 along with co-factor nsp10 is essential for cap-1 structure formation (Figure 1) [12]. Both N7-MTase and 2′-O-MTase use S-adenosyl-l-methionine (SAM) as the methyl donor to methylate the SARS-CoV-2 viral mRNA at the SAM binding site [13]. Stable monomeric protein nsp10 interacts with nsp16 to extend its RNA binding groove and stabilize its SAM binding pocket, both of which are essential for nsp16 MTase activity [14,15,16]. Therefore, the SAM binding sites of nsp14 and nsp16 and the nsp10-nsp16 interface are potential targets for developing highly specific anti-COVID-19 drugs [17]. Several small molecular inhibitors targeting nsp14 or nsp16 have been reported and validated by in vitro experiments, such as Sinefungin, SS148, and WZ16 [14,18,19]. However, most of them are SAM analogs that possess similar scaffolds [20,21]. Thus, further research is still warranted to discover inhibitors with diverse scaffolds and structures.

In this study, we conducted structure-based virtual screening (SBVS) to identify small molecular inhibitors targeting the nsp14 or nsp16 of SARS-CoV-2. A total of 349,000 compounds from the ZINC15 database and 100,000 compounds from the ChemDiv database were collected and screened by filtering steps of the virtual screening workflow (HTVS, SP, XP) of Schrödinger software. A total of 9 and 8 compounds were screened out for the further in vitro experimental validation of N7-MTase and 2′-O-MTase inhibition activities, respectively. Finally, 3 compounds A1–A3 exhibited more than 60% of inhibition against N7-MTase, and 3 compounds B1–B3 exhibited more than 45% of inhibition against 2′-O-MTase. These compounds could be used as potential MTase inhibitors for the future drug design of SARS-CoV-2.

## 2. Results

**Figure 2:**
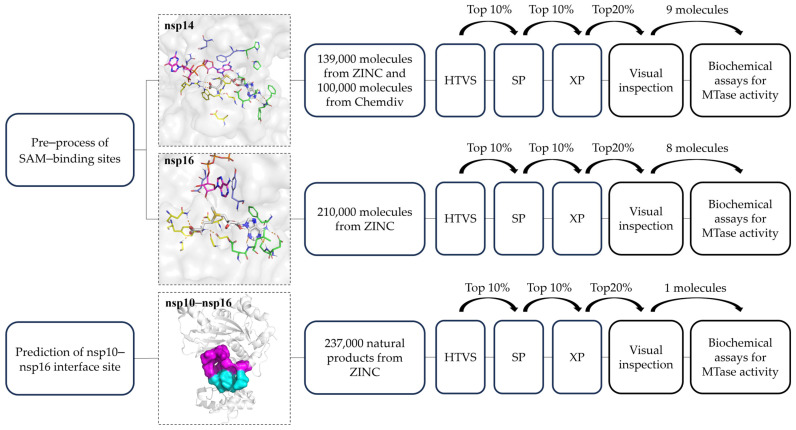
Framework of structure-based virtual screening.

The overall workflow of SBVS for identifying potential SARS-CoV-2 nsp14 or nsp16 inhibitors is presented in Figure 2. In this SBVS process, we first pre-processed the SAM binding sites of nsp14 and nsp16 by analyzing the binding modes of reported SARS-CoV-2 MTase inhibitors, and we predicted the potential binding pocket at the nsp10–nsp16 interface by utilizing the Protein Plus DoGSiteScorer webserver (https://proteins.plus/, accessed on 6 January 2023) [22]. Subsequently, we performed the SBVS successively using Glide HTVS, SP, XP docking, and visual inspection of the binding interactions. Finally, we conducted in vitro assays to validate the MTase inhibition activities of the selected compounds.

### 2.1. Binding Site Processing of SARS-CoV-2 nsp14 and nsp16

The X-ray data analysis of the recently reported crystal structures of SARS-CoV-2 nsp14 and nsp16 was compared and is presented in Tables S1 and S2, and the qualities of these structures were evaluated using the MolProbity webserver (http://molprobity.biochem.duke.edu/index.php, accessed on 20 October 2022) [23] to select a reasonable structure for subsequent SBVS. Consequently, the structures coded with 7R2V and 6WVN [24,25] were selected for nsp14 and nsp16, respectively, based on the rank of structural resolution, Clashscore and MolProbity score, and considering the importance of co-factor SAM/SAH and the substrate for N7 and 2′-O methylation.

**Figure 3:**
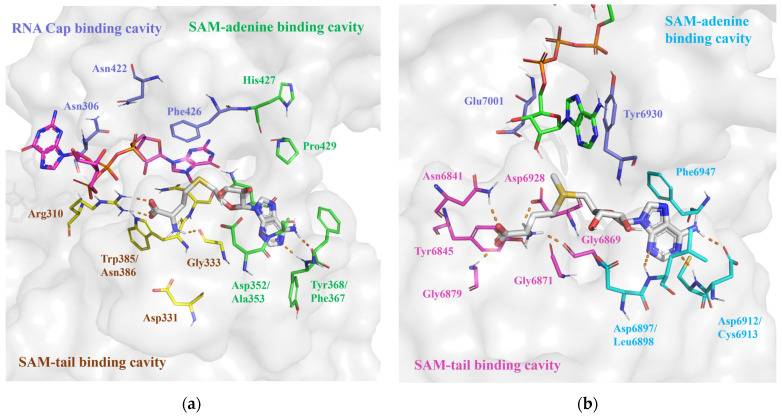
(**a**) Protein surface of SARS-CoV-2 nsp14 SAM binding site (PDB ID: 7R2V). SAH is denoted by white sticks; m7GpppA (align from PDB ID: 7QIF) is denoted by pink sticks; the amino acids of the SAM-adenine, SAM-tail, and RNA cap binding cavity are denoted by green, yellow, and purple sticks, respectively; and hydrogen bonds are denoted by orange dashed lines. (**b**) Protein surface of SARS-CoV-2 nsp16 SAM binding site (PDB ID: 6WVN). SAM is denoted by white sticks; m7GpppA is denoted by green sticks; the amino acids of the SAM-adenine and SAM-tail binding cavity are denoted by cyan and pink sticks, respectively; and hydrogen bonds are denoted by orange dashed lines.

In the SAM binding site of nsp14, the SAM/SAH binding pocket is in close proximity to the RNA binding pocket, and the reported inhibitors are capable of occupying both the SAM and RNA cap binding pockets [26,27,28,29,30,31]. Thus, we pre-processed the nsp14 SAM binding site by dividing it into three parts (Figure 3a): (i) SAM-adenine binding cavity, which includes residues Asp352, Ala353, and Tyr368 that can form hydrogen bond interactions with the adenosine group of the reported SAM analog inhibitors; (ii) SAM-tail binding cavity, containing residues Arg310, Gly333, and Trp385/Asn386 that can form hydrogen bonds with the methionine part of SAM analogs; and (iii) RNA cap binding cavity, including Phe426 that forms a π-π stacking interaction with the base group of the RNA cap. In the subsequent virtual screening, we would select the potential inhibitors that occupy both the SAM-adenine and SAM-tail binding cavities or both the SAM-adenine and RNA cap binding cavities.

The SAM binding site of nsp16 presented a restricted spatial configuration compared with that of nsp14 [17,18,20,32,33,34] because of the clashes between the SAM and RNA cap binding pockets in the presence of m7GpppA in nsp16, leading to an open state of the cap-0 binding pocket [24]. Hence, we divided the nsp16 SAM binding site only into two distinct parts (Figure 3b): (i) SAM-adenine binding cavity, which includes residues Leu6898, Asp6912, and Cys6913 that can form hydrogen bonds with the adenosine group of the reported SAM analog inhibitors; and (ii) SAM-tail binding cavity, containing residues Gly6869, Gly6879, Asn6841, and Asp6928 that can form H-bond interactions with the methionine part of SAM analogs. In the upcoming virtual screenings, we would screen out the potential inhibitors capable of concurrently binding to both the SAM-adenine and SAM-tail binding cavities.

The nsp10–nsp16 interface was identified as a potential target for developing 2′-O-MTase inhibitors through disrupting the nsp10–nsp16 interactions [34]. The potential binding pocket at the nsp10–nsp16 interface was detected using the Protein Plus DoGSiteScorer webserver (https://proteins.plus/, accessed on 6 January 2023) [22,35]. The cavity shown in Figure S1 presented the best region with the highest druggability score of 0.81 and a volume of 589.38 Å^3^ for drug binding. The amino acid residues positioned in the predicted binding pocket are listed in Table S3 and plotted by sticks in Figure S1 using PyMOL (DeLano Scientific, Palo Alto, CA, USA). As can be seen from Figure S1, the amino acid residues in the DoGSiteScorer-predicted binding pocket were consistent with the interacting interface residues (Figure S1) extracted by PDBsum prot-prot analysis [24]. The key residues in the predicted binding pocket were Arg6884, Gln6885, Met7045 of nsp16, and Leu4298, Thr4300, Pro4312, Gly4347, and Tyr4349 of nsp10 (Table S3).

### 2.2. Structure-Based Virtual Screening

For SBVS by glide docking, the receptor grid in the SAM binding site of nsp14 and nsp16 was, respectively, defined as a 30 Å box centered on the O-atom of residue Asn386 and a 35 Å box centered on the 2′-O atom of the m7GpppA substrate. And the receptor grid for SBVS at the nsp10–nsp16 interface was defined as a 30 Å box centered on the O atom of residue Gln6885 in nsp16. The filtering steps of the SBVS workflow (HTVS, SP, XP) in Schrödinger were employed, and visual inspection analysis was also carried out to screen out the potential N7 and 2′-O MTase inhibitors. We first selected the top 10% hits from Glide HTVS for subsequent SP filtering, and then chose the top 10% hits from SP for subsequent XP filtering, and finally the top 20% hits from XP were subjected to visual inspection screening [36,37,38]. In the visual inspection screening step, we used the binding mode and interaction of Sinefungin as the positive control (Figure S2) to screen out compounds with similar or more favorable binding interactions.

#### 2.2.1. SBVS Results of nsp14 Inhibitors

**Figure 4:**
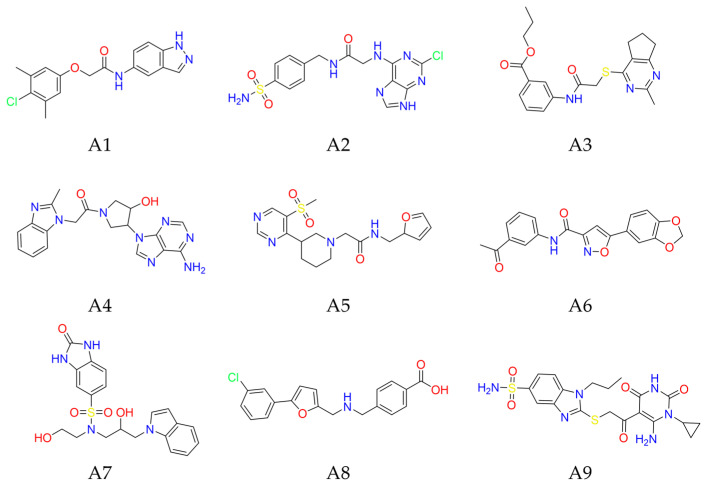
Chemical structures of potential inhibitors A1–A9 targeting nsp14 SAM binding site.

**Figure 5:**
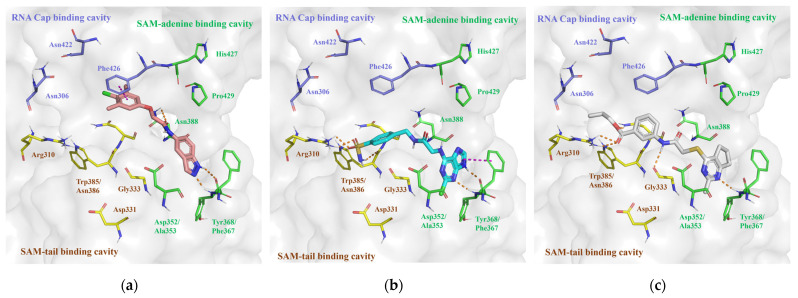
A three-dimensional view of the binding modes of (**a**) A1, (**b**) A2, and (**c**) A3 in the nsp14 SAM binding site. Amino acids in the SAM-adenine, SAM-tail, and RNA Cap binding cavities are denoted by green, yellow, and purple sticks, respectively. The hydrogen bonds are denoted by orange dashed lines, and π-π stacking interactions are denoted by pink dashed lines.

We identified nine potential nsp14 inhibitors A1–A9 (Y207-3841, ZINC000009481760, D306-0032, ZINC000257219502, ZINC000012154664, C226-1222, ZINC000257316872, D665-0380, ZINC000008892924) through multiple rounds of screening (Figure 4, Figure 5, Figures S3 and S7a and Table 1). As shown in Table 1, the molecular weight and logP of these nine compounds ranged from 350 to 480 and 0.3 to 3.8, respectively. Almost all molecules exhibited docking scores less than −8.50 kcal/mol and formed hydrogen bond interactions with Tyr368 in the SAM-adenine binding cavity. Among these nine compounds, seven molecules (A1, A4–A9) occupy both the SAM-adenine and RNA cap binding cavities and engage in π-π interactions with Phe426 in the RNA cap binding cavity. The remaining two molecules (A2–A3) occupy both the SAM-adenine and SAM-tail binding cavities and exhibit a similar binding conformation as SAM and Sinefungin (Figure S2a) that form hydrogen bonds with Arg310 in the SAM-tail cavity. For the top three compounds A1–A3 with high docking scores less than −9.17 kcal/mol (Figure 5, Table 1), the two N-atoms of indazole in compound A1 form hydrogen bonds with Tyr368 in the SAM-adenine cavity and benzene forms a π-π stacking interaction with Phe426 in the RNA cap binding cavity to achieve dual substrate occupancy. The two N-atoms of adenine in compound A2 form two hydrogen bonds with Tyr368, a π-π stacking interaction of adenine with Phe367 in the SAM-adenine cavity, and hydrogen bond interactions of the sulfonyl group with Arg310 and Asn386 in the SAM-tail cavity. The two N-atoms of pyrimidine in compound A3 form hydrogen bonds with Ala353 and Tyr368 in the SAM-adenine cavity and the O-atoms of the ester group form hydrogen bonds with Arg310 and Asn388 in the SAM-tail cavity. Based on the molecular property, docking score, and binding interaction analysis, these nine compounds were selected for further in vitro validations of N7-MTase inhibition activities.

#### 2.2.2. SBVS Results of nsp16 Inhibitors

**Figure 6:**
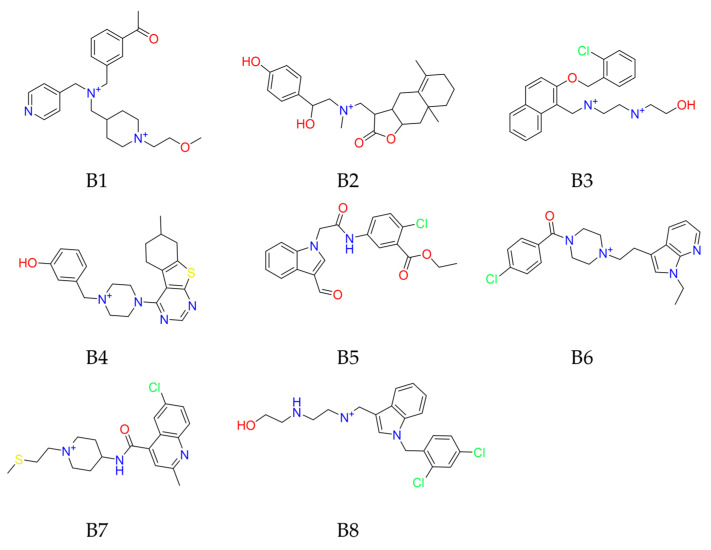
Chemical structures of potential inhibitors B1–B8 targeting nsp16 SAM binding site.

**Figure 7:**
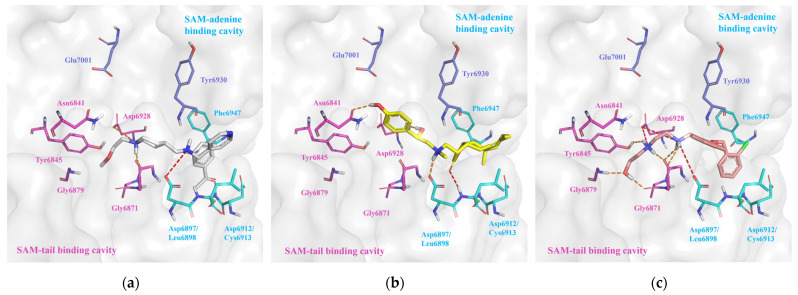
A three-dimensional view of binding modes of (**a**) B1, (**b**) B2, and (**c**) B3 in the nsp16 SAM binding site. Amino acids in the SAM-adenine and SAM-tail cavities are denoted by cyan and pink sticks, respectively. The hydrogen bonds are denoted by orange dashed lines, and salt bridge interactions are denoted by red dashed lines.

In our comprehensive multi-layer virtual screening for nsp16 inhibitors, we focused on two distinct sites, i.e., the SAM binding site and the nsp10–nsp16 interface. For the SAM binding site, we identified eight potential inhibitors B1–B8 (ZINC55183218, ZINC4073149, ZINC95190922, ZINC60349570, ZINC1127559, ZINC65164617, ZINC215527498, ZINC20477654) based on their rank of docking scores and binding interactions (Figure 6, Figure 7, Figures S4 and S7b and Table 2). As shown in Figure 7 and Figure S4, all eight compounds conform to Lipinski’s Rule of Five and occupy both the SAM-adenine and SAM-tail cavities. The top three hits B1–B3 (Figure 7, Table 2) with high docking scores less than −8.30 kcal/mol include positively charged amino groups that form salt bridge interactions with Asp6897 and Asp6928 in the SAM-adenine and SAM-tail cavities, respectively, exhibiting a similar binding conformation as SAM and Sinefungin (Figure S2b). In addition, the positively charged amino groups of these three top molecules form hydrogen bond interactions with Gly6871 and Gly6869 in the SAM binding pocket. Especially, B3 significantly exhibited the greatest number of H-bond and salt-bridge interactions with nsp16, indicating that this molecule might be the most potent nsp16 inhibitor. The in vitro 2′-O-MTase inhibition activities of these eight compounds were tested for further validations and comparison.

For the screening at the nsp10–nsp16 interface, we identified five compounds C1–C5 (ZINC67911283, ZINC67912643, ZINC95785585, ZINC253387786, and ZINC72320248), which are natural products with large molecular weights and multiple hydroxyl groups. As shown in Figures S5, S6 and S7c and Table S4, they form multiple hydrogen bonds with the predicted key amino acids such as Gln6885, Thr6889, Leu7050, Pro4312, and Glu4313 at the nsp10–nsp16 interface, leading to higher docking scores compared with the selected compounds in the SAM binding site of nsp16. However, they exhibited poor drug-like properties because they contain more than five hydrogen bond donors, contravening Lipinski’s Rule of Five for small molecular drugs (Table S4). Thus, we only selected compound C1 with the best binding score and interaction for further in vitro 2′-O-MTase inhibition validation.

#### 2.2.3. ADMET Properties of the Selected Potential MTase Inhibitors

The pharmacokinetic properties of the selected potential MTase inhibitors from SBVS were predicted by pkCSM (https://biosig.lab.uq.edu.au/pkcsm/, accessed on 5 March 2023) [39]. The results presented in Tables S5–S7 indicated that the 9 and 8 selected compounds, respectively, targeting the SAM binding site of nsp14 and nsp16 exhibited moderate absorption, distribution, metabolism, and excretion (ADME) properties, so these 17 compounds were all further validated by in vitro MTase inhibition activity testing, whereas the 5 selected natural products targeting the nsp10–nsp16 interface showed poor absorption and metabolism properties, and we only chose compound C1 with the best ADMET properties for further in vitro validation.

### 2.3. Biochemical Assays for MTase Inhibition Activity

The selected potential inhibitors of nsp14 and nsp16 were validated by in vitro methyltransferase activity testing under a concentration of 50 µM. Consequently, for nsp14, the positive control Sinefungin showed 90.91% of inhibition under 25 µM, and among the 9 selected compounds, 3 (A1–A3) of them exhibited N7-MTase inhibitory rates higher than 60% (Table 1) and were also the top 3 hits in the SBVS. Notably, for nsp16, the positive control Sinefungin showed 86.34% of inhibition under 50 µM, and of the 8 tested compounds binding to the nsp16 SAM site, the top 3 hits (B1–B3) in the virtual screening showed the most potent 2′-O-MTase inhibition activities with inhibitory rates higher than 45% (Table 2), whereas compound ZINC67911283 targeting the nsp10–nsp16 interface only showed a 2′-O-MTase inhibitory rate of 3.72% probably due to its poor drug-like property. As a result, we successfully identified six SARS-CoV-2 MTase inhibitors with moderate activities.

### 2.4. Molecular Dynamics Simulation

In order to investigate the conformational stability and dynamic features of the six hits, we performed 100 ns MD simulations at 310 K for each system after 700 ps equilibrations. The dynamic parameters such as the root-mean-square deviation (RMSD) of the nsp14 and nsp16 protein backbone and ligands were plotted as a function of time (Figure S8), and the critical distances (Figures S9–S14) that describe the binding interactions between ligands and protein were also analyzed to validate the stability of the binding pose and interactions.

The RMSD trajectories for the protein backbone and ligands shown in Figure S8 indicated that the MD simulations for the six systems reached convergence at around 40 ns, and the dynamic conformations of the proteins and ligands were stable after 40 ns. The critical distance trajectories shown in Figures S9–S14 revealed that the binding poses and key interactions of the six hits in the nsp14/nsp16 protein were almost maintained. For nsp14, all three hits (A1–A3) preserved the conformation of dual substrate occupancy and maintained the hydrogen bond interaction with Tyr368 in the SAM-adenine cavity (Figures S9–S11). For nsp16, the hydrogen bond interactions between the three hits (B1–B3) and the amino acids in the SAM-tail cavity were maintained. Notably, the salt bridge interactions between the positively charged amino groups of the three hits and amino acids Asp6897 or Asp6897 in nsp16 were retained (Figures S12–S14). These results demonstrated the stable conformational stability and dynamic features of the final six hits in the nsp14/nsp16 protein.

## 3. Discussion

SARS-CoV-2 and its mutants have caused millions of deaths globally. Though effective vaccines and antiviral drugs have been developed, there is evolving resistance to these vaccines and drugs. Consequently, it is desirable to develop antivirals targeting enzymes central to the life cycle of SARS-CoV-2. The methyltransferases nsp14 and nsp16 of SARS-CoV-2 are such enzymes that use SAM as a cofactor to methylate the N7 and 2′-O positions of the 5′-end of viral mRNA to evade the host immune response [40]. Unlike SARS-CoV-2 M^pro^ and RdRp enzymes [41,42] that were developed as investigational drugs through similarities to other viruses, the MTase of nsp14 and nsp16 have seen little research [43]. In addition, most of the reported small-molecule inhibitors of nsp14 or nsp16 are SAM analogs, whose hydrophilicity hinders their ability to cross cell membranes [40]. Thus, it is desirable to discover more nsp14 and nsp16 inhibitors with diverse scaffolds.

In this study, we utilized SBVS to screen out potential small molecular MTase inhibitors of SARS-CoV-2 based on the high-resolution co-crystal structure of nsp14 (PDB ID: 7R2V) and nsp16 (PDB ID: 6WVN). SBVS on the SAM binding sites of the nsp14/nsp16 and nsp10–nsp16 interface was subsequently performed using Glide HTVS, SP, XP docking, and visual inspection of the binding interactions through Schrödinger software. Finally, 9, 8, and 1 compounds targeting SAM binding sites of the nsp14/nsp16 and nsp10–nsp16 interface were screened and validated by in vitro biochemical assays for MTase inhibition activity. The results revealed that 3 potential nsp14 inhibitors A1–A3 exhibited N7-MTase inhibition rates higher than 60%, and 3 compounds B1–B3 targeting the nsp16 SAM binding site showed 2′-O-MTase inhibition rates higher than 45%. Interestingly, the top 3 compounds from SBVS of the nsp14 SAM binding site and the top 3 compounds of the nsp16 SAM binding site were also experimentally validated as the most potent MTase inhibitors, indicating the rationality of our SBVS strategy. Notably, compounds B1–B3 are not SAM analogs but exhibit similar binding modes to SAM, providing new scaffolds for the further study of nsp16 inhibitors. In addition, in the molecular dynamics simulations, we further verified the binding stability of the six identified compounds to their target receptor, illustrating their sustained interaction throughout the simulation trajectories.

There are still limitations in this study. Firstly, the MTase inhibition activities of the top six hits for nsp14 and nsp16 are weak compared with the SAM analog Sinefugin (90.91% inhibition of N7-MTase activity under 25 µM, and 86.34% inhibition of 2′-O-MTase activity under 50 µM). Secondly, the MTase inhibition rate of the tested compound C1 targeting the nsp10–nsp16 interface is only 3.7%. These limitations of SBVS results are probably attributed to the large size of the SAM and interface pockets and the lack of knowledge of crucial residues that determine inhibition potency and selectivity. These factors make the nsp14 and nsp16 targets challenging for docking [43]. In addition, the potency of the nsp10–nsp16 interface as a drug target still requires further experimental validations. Nevertheless, our findings could provide candidates with new scaffolds for the further development of SARS-CoV-2 MTase inhibitors.

## 4. Materials and Methods

### 4.1. Processing of nsp14 and nsp16 Structures

The crystal structures of SARS-CoV-2 nsp14 (PDB ID: 7R2V) and SARS-CoV-2 nsp10-nsp16 complexes (PDB ID: 6WVN) for SBVS were downloaded from Protein Data Bank (https://www.rcsb.org/, accessed on 20 October 2022). Prior to SBVS, the structures of SARS-CoV-2 nsp14 and nsp10–nsp16 complexes were prepared using the protein preparation wizard module of Schrödinger software (Release 2019-2, Schrödinger LLC, New York, NY, USA). The protein preparations, including protonation—state adjustment, water removal, disulfide bonds, hydrogen atom and missing heavy atom addition, and structural minimization, were performed by the Maestro module of Schrödinger software.

### 4.2. Processing of Small Molecules

For SARS-CoV-2 nsp14, we obtained a dataset of 139,000 ligands from the ZINC15 database for virtual screening. These compounds were selected according to the physicochemical properties of the reported active molecules, whose molecular weights and logP values ranged from 375 to 500 and −1 to 1, respectively [17,26,27,29,31,43,44]. Additionally, we selected an extra dataset of 100,000 molecules from ChemDiv (https://www.chemdiv.com/catalog/diversity-libraries, accessed on 30 October 2022/100k Diverse Compounds Pre-Plated Set) to search for inhibitors with diverse scaffolds.

Regarding the SARS-CoV-2 nsp16 SAM binding site, we selected the ligands with a molecular weight of 400 and logP of 4 from the ZINC15 database (https://zinc.docking.org, accessed on 25 October 2022) according to the physicochemical properties of the reported active nsp16 inhibitors [17,18,20,24] and ended up with 210,000 ligands to perform the virtual screening. For the potential binding pocket at the nsp10–nsp16 interface, 237,000 natural products retrieved from the biogenic subset of ZINC15 were collected for SBVS following the virtual screened database used by Mohammad et al. [34].

The downloaded compounds were collected in simplified molecular-input line-entry system (SMILES) format. Then, the LigPrep panel in Maestro was employed for ligand preprocessing which includes (i) an OPLS_2005 force field, (ii) no change for ionization, (iii) a desalt option, (iv) chirality determination from the 3D structure, (v) the generation of one low energy conformer at most per ligand, and (vi) an output in SDF format. The generated 3D conformers of all compounds were subjected to SBVS.

### 4.3. Structure-Based Virtual Screening

The SBVS parameters in the SAM binding site of SARS-CoV-2 nsp14 and nsp16 were determined by redocking the substrate SAH and SAM into nsp14 and nsp16, respectively, to resume the binding mode and interactions of SAH and SAM in the co-crystal structures of nsp14 (PDB ID: 7R2V) and nsp16 (PDB ID: 6WVN). Consequently, the receptor grids for the SBVS of nsp14 and nsp16 were defined as a 30 Å box centered on the O-atom of residue Asn386 and a 35 Å box centered on the 2′-O atom of the m7GpppA substrate, respectively.

Considering that there is no reported co-crystallized ligand that binds to the SARS-CoV-2 nsp10–nsp16 interface, the SBVS parameters were determined by docking the reported potential natural product inhibitor Genkwanin-6-C-beta-glucopyranoside, which was identified by virtual screening against the nsp10 interface [34], into the predicted binding site of the nsp10-nsp16 interface with different box sizes and docking centers near the key residues using the Glide module of Schrödinger software. As a result, the receptor grid for SBVS was defined as a 30 Å box centered on the O atom of key residue Gln6885 in chain A, because the docking of Genkwanin-6-C-beta-glucopyranoside using this grid generated the best binding affinity and most favorable interactions with nsp10–nsp16 interface residues. All other parameters were kept as the default in Schrödinger.

The virtual screening workflow (HTVS, SP, and XP) of Schrödinger (Maestro 11.6.013) was utilized for the SBVS. Initially, the Glide High-Throughput Virtual Screening (HTVS) mode was employed for the preliminary screening phase, and the top 10% hits with the highest binding scores from HTVS were used for the subsequent filtering by the Glide Standard Precision (SP) mode. Then, the top 10% hits from SP were utilized for the next round of screening by the Glide Extra Precision (XP) methodology, and the top 20% hits from XP were subjected to the visual inspection screening [36,37,38]. Finally, the potential small-molecule inhibitors exhibited similar or more favorable binding interactions compared with Sinefungin, which were selected and purchased from TargetMol (https://www.tsbiochem.com/, accessed on 20 February 2023).

### 4.4. Biochemical Assays

#### 4.4.1. RNA Substrate Preparation

The RNA substrate of 5′-terminal 259 nucleotides (ATP as the viral initial nucleotide) of the SARS-CoV-2 genome (uncapped SARS-CoV-2 RNA) was in vitro-transcribed from PCR products by using the MEGAscript Kit (Ambion, Austin, TX, USA) as described in our previous work [45]. By using the vaccinia virus capping enzyme system (Novoprotein, Suzhou, China), the transcribed RNAs were capped/methylated to form the GpppG/A and m7GpppG/A-capped RNAs in the presence or absence of the methyl donor SAM. Primers used for the synthesis of RNA substrates were as follows—Forward-5′: TAATACGACTCACTATTAGATTAAAGGTTTATACCTTCCCAGG, Reverse-5′: CTTTCGGTCACACCCGGAC.

#### 4.4.2. Protein Expression and Purification

The coding sequences of SARS-CoV nsp10, 16; SARS-CoV-2 nsp10, 14, 16; and mutants were cloned into a pET32a vector with the His tag. *E. coli* BL21 (DE3) cells were transformed with the respective plasmid and the recombinant protein was induced with 0.4 mM isopropyl β-d-thiogalactopyranoside (IPTG) at 16 °C for 12–16 h. The cells were harvested by centrifugation, and the pellets were resuspended in lysis buffer (50 mM Tris–HCl, pH 8.0, 300 mM NaCl, 10% glycerol, and 5 mM MgCl_2_). The cells were then disrupted by a high-pressure cracker (UH-24, Union-biotech, Shanghai, China), and cell debris was removed by centrifugation. pET32a-His6-nsp10, 14, and 16 were purified with nickel-nitrilotriacetic acid (Ni-NTA, Shanghai, China) resin (GenScript, Piscataway, NJ, USA) as described previously [45,46].

#### 4.4.3. Radioactive Biochemical Assays for MTase Activity

SARS-CoV-2 nsp14 and nsp16/10 inhibition assays of the final selected compound were carried out in a 30 μL reaction mixture [40 mM Tris-HCl (pH 7.5), 2 mM MgCl_2_, 2 mM DTT, 40 units RNase inhibitor, 0.01 mM SAM], with 0.5 μCi of S-adenosyl [methyl-3H] methionine (67.3 Ci/mmol, 0.5 μCi/mL), 1 μg of purified proteins, and 2 μg of m7GpppA RNA substrates at 37 °C for 1.5 h. The 3H-labeled product was isolated in small DEAE-Sephadex columns and quantitated by liquid scintillation.

### 4.5. Molecular Dynamics Simulations

The molecular dynamics (MD) simulation was performed by the Amber 18 software package installed on a Linux platform. The high-performance server cluster on the platform was composed of two Intel^®^ Xeon^®^ Platinum 8176 CPU (Intel, Santa Clara, CA, USA) processors accelerated by two NVIDIA Tesla V100 SXM2 GPUs (NVIDIA, Santa Clara, CA, USA). The FF14SB force field and GAFF2 force field were applied to proteins and ligands, respectively. Throughout the simulation, the coordination distances of the zinc ions including ZN-S (2.40 Å) and ZN-N (2.10 Å) were restrained by a binding constant of 25 kcal mol^−1^ Å^−2^ [47]. Next, the charges of the 6 compounds were calculated and assigned by Gaussian16 and the Restrained Electro Static Potential (RESP) module in Amber18 software packages. The complexes were then solvated into the pre-equilibrated TIP3P water under periodic boundary conditions using a cubic box model with a 15 Å buffer distance, and 15 and 7 Cl^-^ ions were added to neutralize the system of nsp14 and nsp16, respectively. Subsequent energy minimizations and MD equilibrium simulations followed a similar protocol to our previous studies [48,49]. We first performed 2500 steps of steepest descent minimization followed by a 2500-cycle conjugate gradient minimization by restraining the protein and ligand with a force constant of 50 kcal·mol^−1^·Å^−2^. Then, we performed a 100 ps NVT equilibration simulation (T = 10 K) followed by another NPT (P = 1 atm) equilibration simulation with the restraint force constant gradually decreased to 25 kcal·mol^−1^·Å^−2^. Next, we performed a 200 ps temperature annealing NVT simulation (T was raised from 10 K to 310 K) and a 100 ps NPT simulation with the restraint force constant reduced to 10 kcal·mol^−1^·Å^−2^. Then, we performed two sequential 100 ps NPT simulations with reduced restraint force constants of 1 and 0.1 kcal·mol^−1^·Å^−2^, respectively. Finally, a 100 ns production NPT (T = 310 K and P = 1 atm) simulation was carried out without any restraints. In all MD simulations, the SHAKE algorithm was utilized to constrain the bond length [50], and a 10 Å cutoff was used for both short-range and van der Waals (vdW) interactions. The integration was kept with a 2-fs step. The minimizations and equilibrations were carried out by the sander module, and the three independent 100 ns production MD simulations were performed by the PMEMD.CUDA module in Amber18 [51]. The trajectories were analyzed using the CPPTRAJ package in Amber18 [52].

## 5. Conclusions

In summary, SBVS was performed to explore potential SARS-CoV-2 nsp14 and nsp16 methyltransferase inhibitors. The virtual screening workflow (HTVS, SP, XP) in Schrödinger software combined with visual inspection of the binding interactions were used for our screening of 349,000 compounds from the ZINC database and 100,000 compounds from the ChemDiv database. Consequently, the top 9 and 8 hits targeting SAM binding sites of nsp14 and nsp16, respectively, with the best binding affinities and most favorable interactions were filtered out for further in vitro MTase inhibition activity validation. Finally, three potential inhibitors A1–A3 of nsp14 were identified which exhibited over 60% of inhibition of N7-MTase activity under a concentration of 50 µM. Moreover, three molecules B1–B3 surpassing 45% of inhibition of 2′-O-MTase activity at the concentration of 50 µM were identified as potential inhibitors for nsp16. These findings could provide potential lead compounds for the rational drug design of SARS-CoV-2.

## Figures and Tables

**Table 1:** The SBVS and in vitro validation results of potential inhibitors targeting nsp14 SAM binding site.

Code	Compound	Molecular Weight	LogP	Docking Score (kcal/mol)	H-Bond Interaction	π-π Stacking Interaction	Inhibition Rate (%) ^2^
A1	Y207-3841	329.78	3.78	−10.40	Tyr368(2) ^1^, Asn388(2)	Phe426	68.40
A2	ZINC000009481760	395.83	0.38	−9.80	Arg310(2), Ala353, Tyr368(2), Trp385	Phe367	64.25
A3	D306-0032	385.48	3.78	−9.17	Arg310(2), Asn386, Ala353, Tyr368	\	69.15
A4	ZINC000257219502	392.42	0.51	−9.15	Ala353, Tyr368(2)	Phe426	47.16
A5	ZINC000012154664	378.45	0.97	−8.95	Ala353, Tyr368, Asn388	Phe426	20.79
A6	C226-1222	350.33	2.70	−8.85	Ala353, Tyr368, Asn386	Phe426	46.92
A7	ZINC000257316872	430.49	0.86	−8.79	Gly333, Tyr368, Asn388	Phe426	4.07
A8	D665-0380	378.25	1.42	−8.69	Tyr368	Phe426	33.39
A9	ZINC000008892924	478.56	0.84	−8.58	Arg310, Gly333, Asn388	Phe426	11.20

^1^ This represents the compound that forms two hydrogen bonds with the same amino acid. ^2^ The inhibition rate was calculated by taking the average of three parallel experiments at 50 µM.

**Table 2:** The SBVS and in vitro validation results of potential inhibitors targeting nsp16 SAM binding site.

Code	Compound	Molecular Weight	LogP	Docking Score (kcal/mol)	H-Bond Interaction	Salt Bridge Interaction	Inhibition Rate (%) ^2^
B1	ZINC55183218	397.6	2.50	−8.70	Gly6871, Cys6913	Asp6897, Asp6928,	49.06
B2	ZINC4073149	400.5	3.69	−8.60	Leu6898, Asp6928	Asp6897	48.82
B3	ZINC95190922	386.9	3.52	−8.30	Gly6869(2) ^1^, Ala6870, Gly6871, Gly6879, Asp6928	Asp6897, Asp6928	54.91
B4	ZINC60349570	395.5	4.63	−8.26	Leu6898, Cys6913, Lys6968, Asp6928	Asp6897	0
B5	ZINC1127559	384.8	3.31	−8.06	Asn6841, Asp6897, Cys6913, Tyr6930	\	26.82
B6	ZINC65164617	397.9	3.42	−7.76	Asp6873, Asp6897	Asp6897	0.78
B7	ZINC215527498	378.9	3.74	−7.57	Tyr6830, Gly6871, Asp6897, Cys6913	Asp6897	0
B8	ZINC20477654	393.3	3.11	−7.51	Gly6869, Ala6870, Gly6879, Asp6928(2)	Asp6928	0

^1^ This represents the compound that forms two hydrogen bonds with the same amino acid. ^2^ The inhibition rate was calculated by taking the average of three parallel experiments at 50 µM.

## Data Availability

All data are included in the article and Supplementary Materials.

## Supplementary Materials

The following supporting information can be downloaded at https://www.mdpi.com/article/10.3390/molecules29102312/s1. Table S1: The X-ray data analysis of the SRAS-CoV-2 nsp14 structures reported in the PDB database. Table S2: The X-ray data analysis of the SRAS-CoV-2 nsp10–nsp16 complexes reported in the PDB database. Figure S1: (a) The DoGSiteScorer-predicted binding pocket (shown by surface) at the nsp10–nsp16 interface (6WVN) with a druggability score of 0.81 and volume of 589.38 Å^3^; (b) schematic of the non-bonded interactions between interface residues of nsp16 (Chain A) and nsp10 (Chain B) extracted by PDBsum prot-prot analysis. Table S3. The amino acid residues positioned in the DoGSiteScorer predicted binding pocket of nsp10–nsp16 interface (key residues are labeled by bold). Figure S2: (a) A three-dimensional view of the binding interactions of Sinefungin in the nsp14 SAM binding site; (b) a three-dimensional view of the binding interactions of Sinefungin in the nsp16 SAM binding site. Figure S3: A three-dimensional view of the binding interaction of A4–A9 (a–f) in the nsp14 SAM binding site. Figure S4: A three-dimensional view of the binding interaction of B4–B8 (a–e) in the nsp16 SAM binding site. Figure S5: Chemical structures of nsp10–nsp16 interface site inhibitors. Figure S6: A three-dimensional view of the binding interaction of C1–C5 (a–e) at the nsp10–nsp16 interface. Table S4: Molecular docking results of nsp10–nsp16 interface site inhibitors. Figure S7: Docking score distribution of glide HTVS, SP, and XP docking steps in VS of compounds targeting the SAM binding sites of nsp14 (a) and nsp16 (b) and the nsp10–nsp16 interface (c). Table S5: Predicted ADMET properties of SARS-CoV-2 nsp14 inhibitors by pkCSM. Table S6: Predicted ADMET properties of SARS-CoV-2 nsp16 inhibitors by pkCSM. Table S7: Predicted ADMET properties of SARS-CoV-2 nsp10–nsp16 interface inhibitors by pkCSM. Figure S8: The RMSD of protein backbone (a) and ligands (b) as a function of time for the 6 hits bound to SARS-CoV-2 nsp14 and nsp16 SAM binding sites in the 100 ns MD simulations. Figures S9–S14: The predicted 3D binding mode of compounds (A1–A3, B1–B3) bound to SARS-CoV-2 nsp14/nsp16, and the distances that describe the binding interactions between the ligand and protein as a function of time in the 100 ns MD simulation.
