# Supplementary material for: Structure-Based Virtual Screening for Methyltransferase Inhibitors of SARS-CoV-2 nsp14 and nsp16
Source: Molecules. 2024 May 15;29(10):2312. doi: 10.3390/molecules29102312 (PMC11124212; doi:10.3390/molecules29102312)
Supplement: Supplementary file 1 [file molecules-29-02312-s001.zip › Supporting Materials.pdf]

Table S1. The x-ray data analysis of the SRAS-CoV-2 nsp14 structures reported in the PDB database.

PDB ID	Revolution (Å)	Released	In Complex With	Clashscore [1]	MolProbity score [1]
7TW9	1.41	2022-09-07	TELSAM and Sinefungin	5.34	1.78
7TW8	1.55	2022-09-07	TELSAM and SAH	6.62	1.86
7TW7	1.62	2022-09-07	SAM	9.81	1.98
**7R2V**	**2.53**	**2022-03-09**	**SAH**	**3.05**	**2.05**
5SLS	2.29	2022-03-16	inhibitor	3.73	2.07
7QIF	2.53	2022-02-02	m7GpppG	2.06	1.52
7QGI	1.65	2022-01-26	\	2.22	1.32
7DIY	2.693	2021-05-19	\	9.61	2.23

[1] Lower Clashscore and MolProbity score indicate higher quality.

Table S2. The x-ray data analysis of the SRAS-CoV-2 nsp10–nsp16 complexes reported in the PDB database.

PDB ID	Revolution (Å)	Released	In Complex With	Clashscore [1]	MolProbity score [1]
7R1U	2.50	2022-06-29	m7GpppA and WZ16	3.02	1.09
7R1T	2.70	2022-06-29	SS148	2.88	1.13
7ULT	1.90	2022-04-13	/	2.36	1.18
7LW3	2.30	2021-05-05	m7GpppA and SAH	6.35	1.77
7LW4	2.50	2021-05-05	SAH	2.68	1.42
7JJY	2.05	2020-09-16	m7GpppA and SAM	8.25	2.59
7JPE	2.18	2020-08-26	m7GpppA and SAM	1.83	1.16
7BQ7	2.37	2020-06-17	SAM	7.42	2.01
7C2J	2.80	2020-05-27	SAM	3.60	1.51
**6WVN**	**2.00**	**2020-05-13**	**m7GpppA and SAM**	**1.72**	**1.08**
6YZ1	2.40	2020-05-13	Sinefungin	7.34	1.65
6WQ3	2.10	2020-05-06	m7GpppA and SAH	1.92	1.05
6WKQ	1.98	2020-04-16	Sinefungin	2.27	1.20
6W75	1.95	2020-03-25	SAM	3.45	1.33
6W61	2.00	2020-03-25	SAM	3.71	1.54
6W75	1.95	2020-03-25	SAM	3.45	1.33
6W4H	1.80	2020-03-18	SAM	3.50	1.23

[1] Lower Clashscore and MolProbity score indicate higher quality.

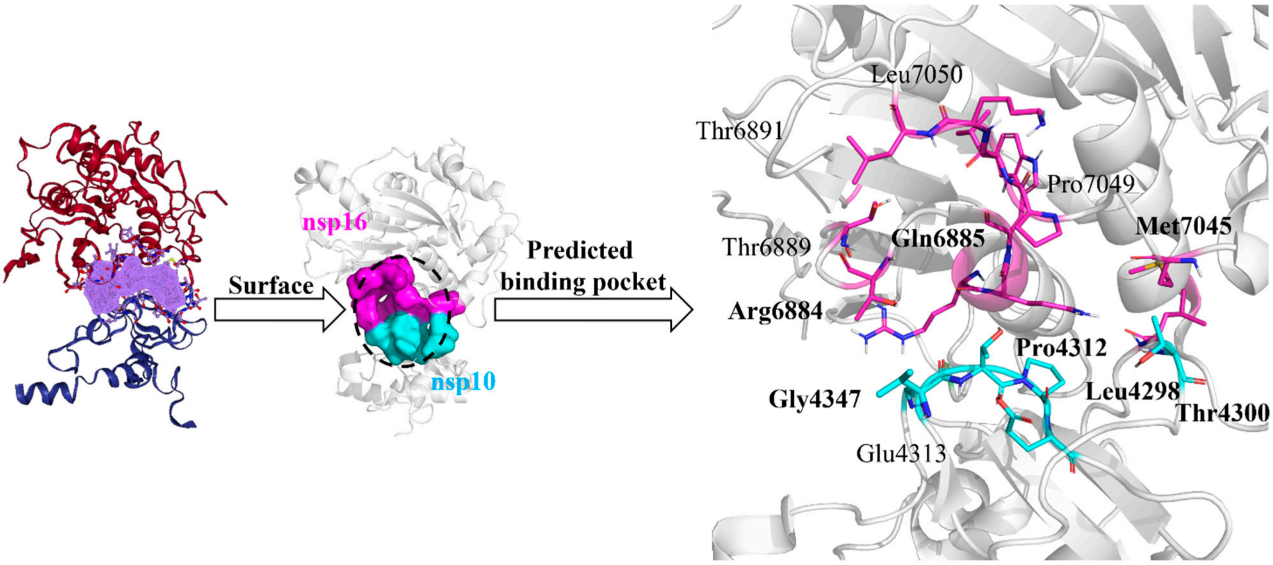

(**a**)

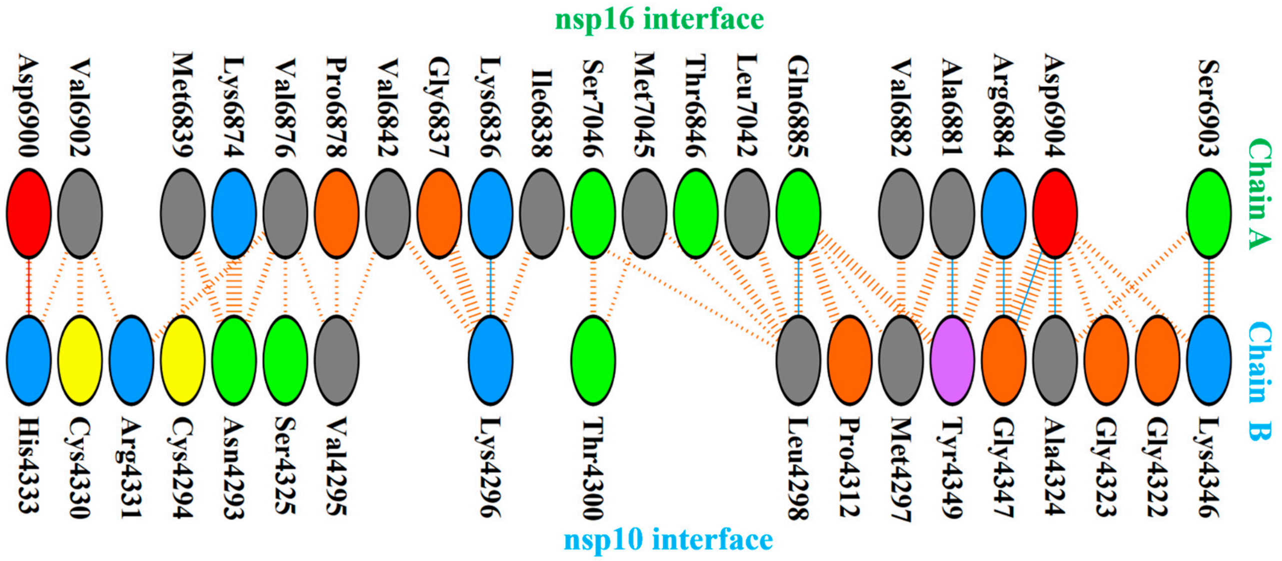

(**b**)

Figure S1. (**a**)The DoGSiteScorer predicted binding pocket (shown by surface) in the nsp10–nsp16 interface (6WVN) with a druggability score of 0.81 and volume of 589.38 Å^3. The nsp16 and nsp10 are shown by light and dark gray cartoon respectively. The amino acid residues of nsp16 and nsp10 positioned in the predicted binding pocket are shown by pink and cyan sticks respectively. The DoGsiteScorer predicted key residues in the pocket are labeled by bold characters. (**b**) Schematic of the non-bonded interactions between interface residues of nsp16 (Chain A) and nsp10 (Chain B) extracted by PDBsum prot–prot analysis.

Table S3. The amino acid residues positioned in the DoGSiteScorer predicted binding pocket of nsp10–nsp16 interface (key residues are labeled by bold).

SARS-CoV-2	Residues in the predicted binding pocket
nsp16 (Chain A)	Leu6883, **Arg6884, Gln6885,** Trp6886, Leu6887, Pro6888, Thr6889, Gly6890, Thr6891, Leu6893, Asp6906, Met7045, Phe7048, Pro7045, Leu7050
nsp10 (Chain B)	**Leu4298,** Cys4299, **Thr4300,** His4301, Thr4302, Gly4303, Val4310, Thr4311, **Pro4312,** Glu4313, Ala4314, Ser4320, **Gly4347,** Cys4343, **Tyr4349**

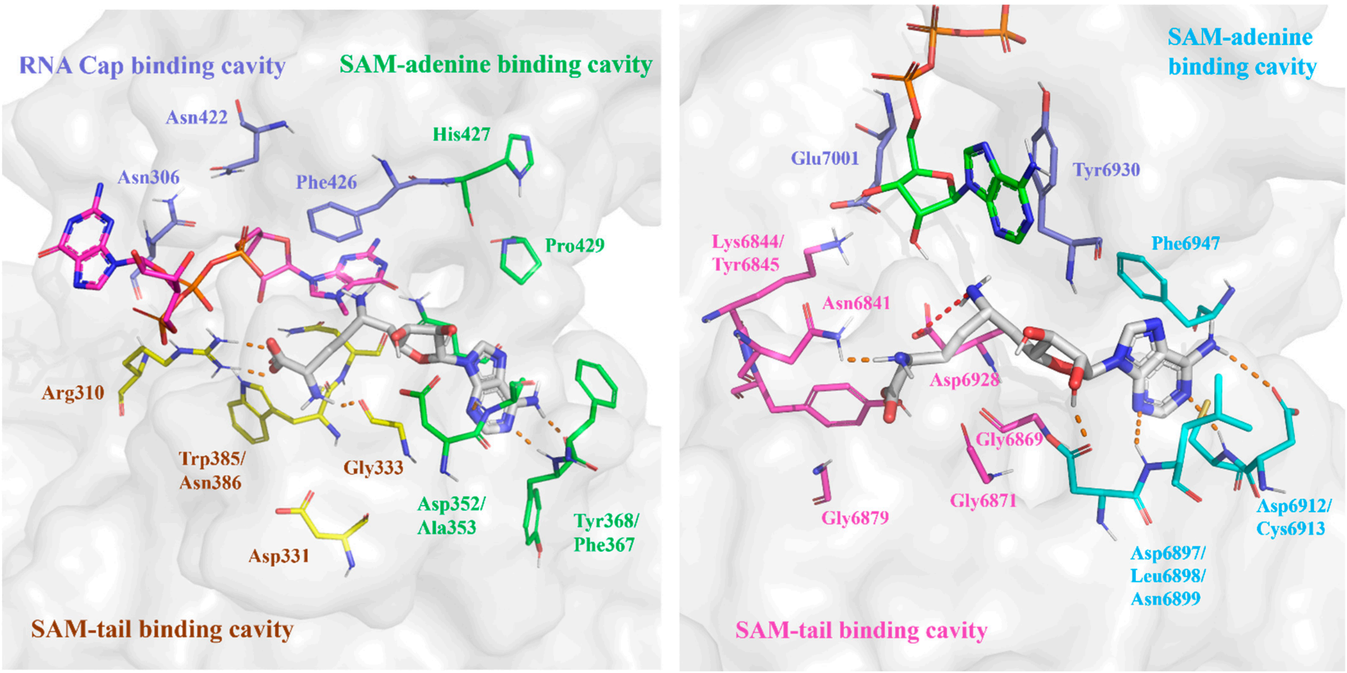

(**a**) (**b**)

Figure S2. (**a**) A three–dimensional view of the binding interactions of Sinefungin in the nsp14 SAM binding site. The amino acids of the SAM–adenine, SAM–tail and RNA cap binding cavity are displayed by green, yellow, and purple sticks respectively, and hydrogen bonds are displayed by orange dashed line. (**b**) A three–dimensional view of the binding interactions of Sinefungin in the nsp16 SAM binding site. The amino acids of the SAM–adenine and SAM–tail binding cavity are displayed by cyan and pink sticks respectively, hydrogen bonds are displayed by orange dashed lines, and salt bridge interactions are displayed by red dashed lines.

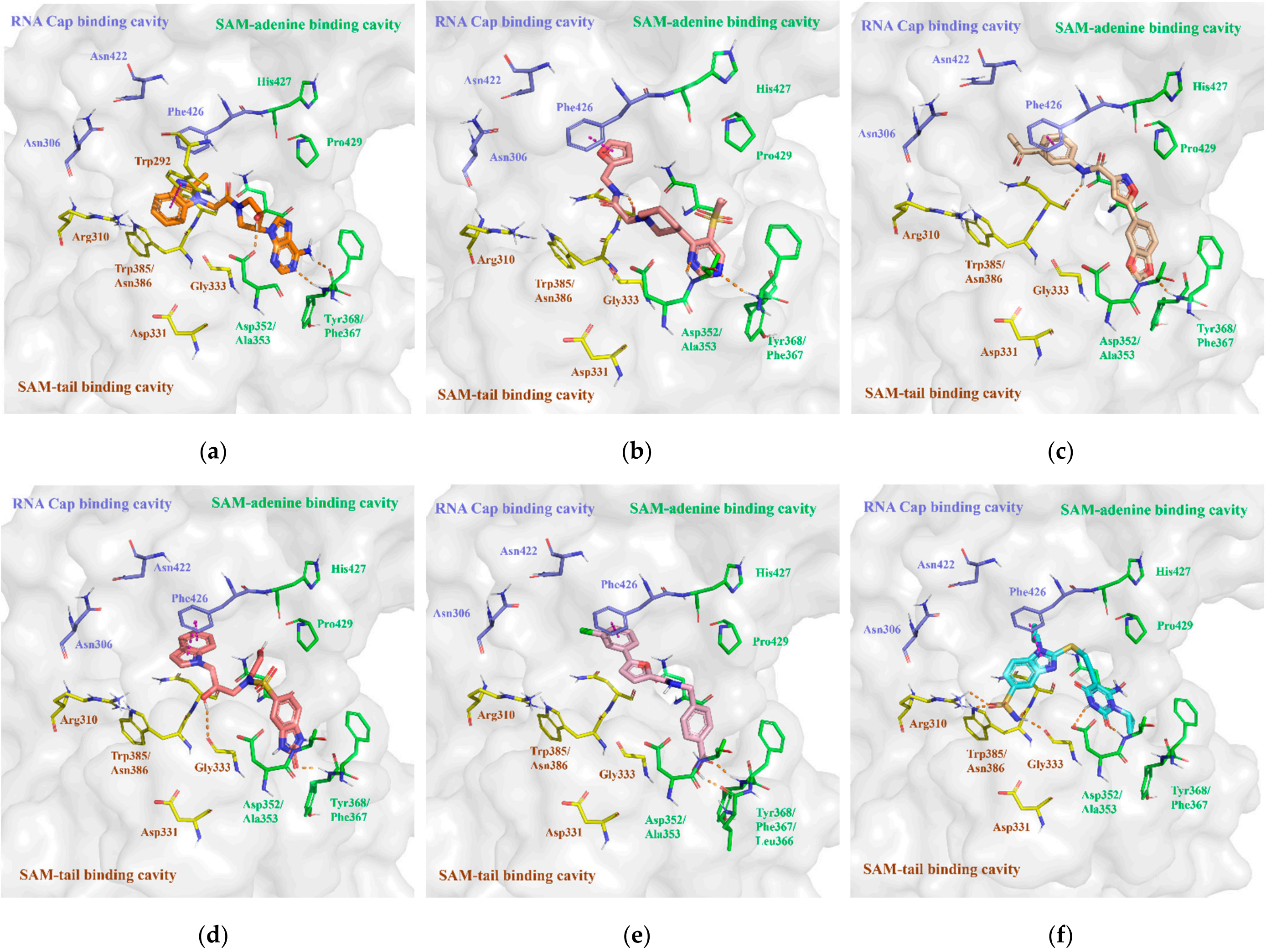

Figure S3. A three–dimensional view of the binding interactions of A4–A9 (**a-f**) in the nsp14 SAM binding site. The amino acids of the SAM–adenine, SAM–tail and RNA cap binding cavity are displayed by green, yellow, and purple sticks respectively, hydrogen bonds are displayed by orange dashed lines, and π-π stacking interactions are displayed by pink dashed lines.

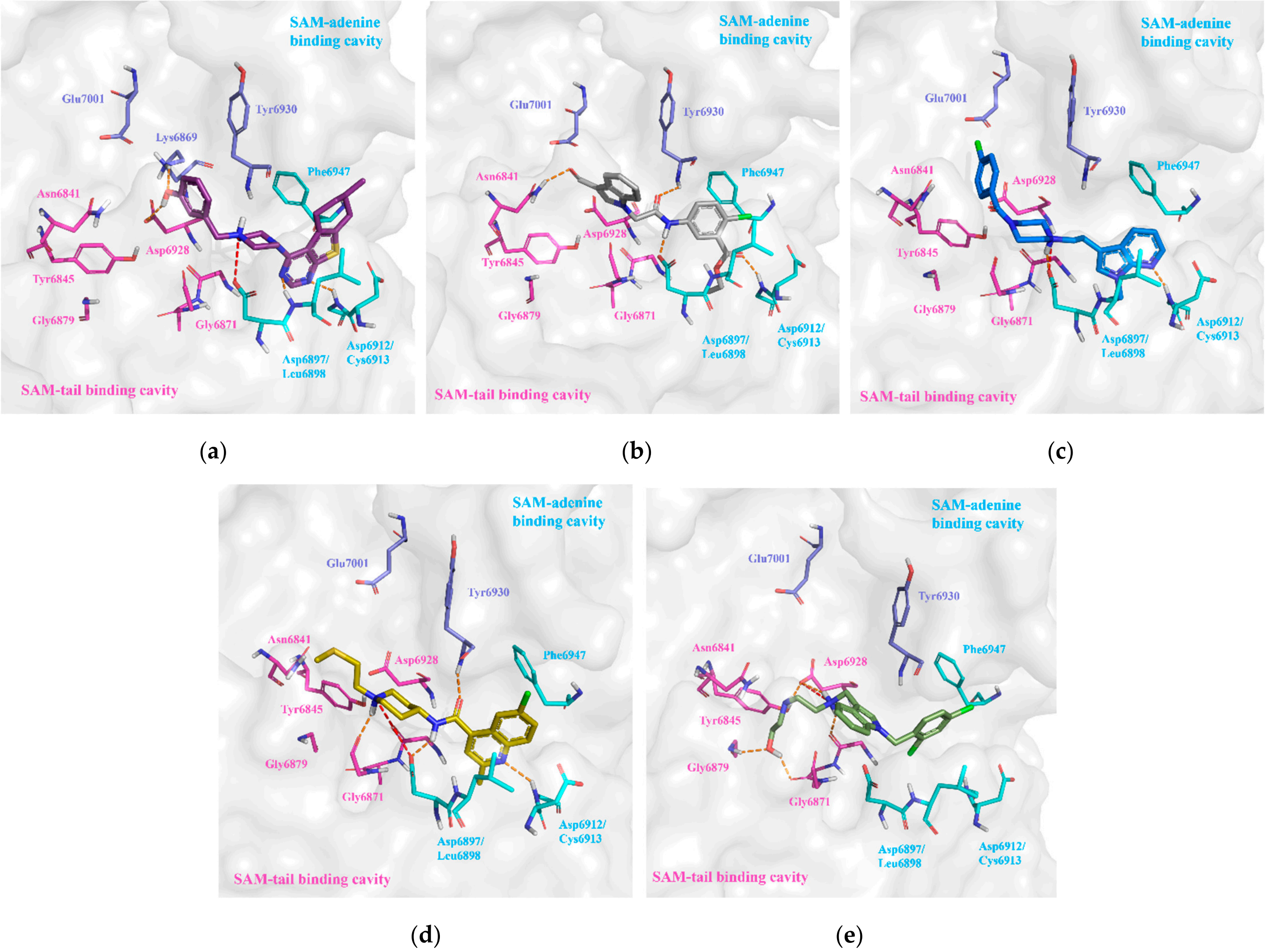

Figure S4. A three–dimensional view of the binding interactions of B4-B8 (**a-e**) in the nsp16 SAM binding site. The amino acids of the SAM–adenine and SAM–tail binding cavity are displayed by cyan and pink sticks respectively, hydrogen bonds are displayed by orange dashed lines, and salt bridge interactions are displayed by red dashed lines.

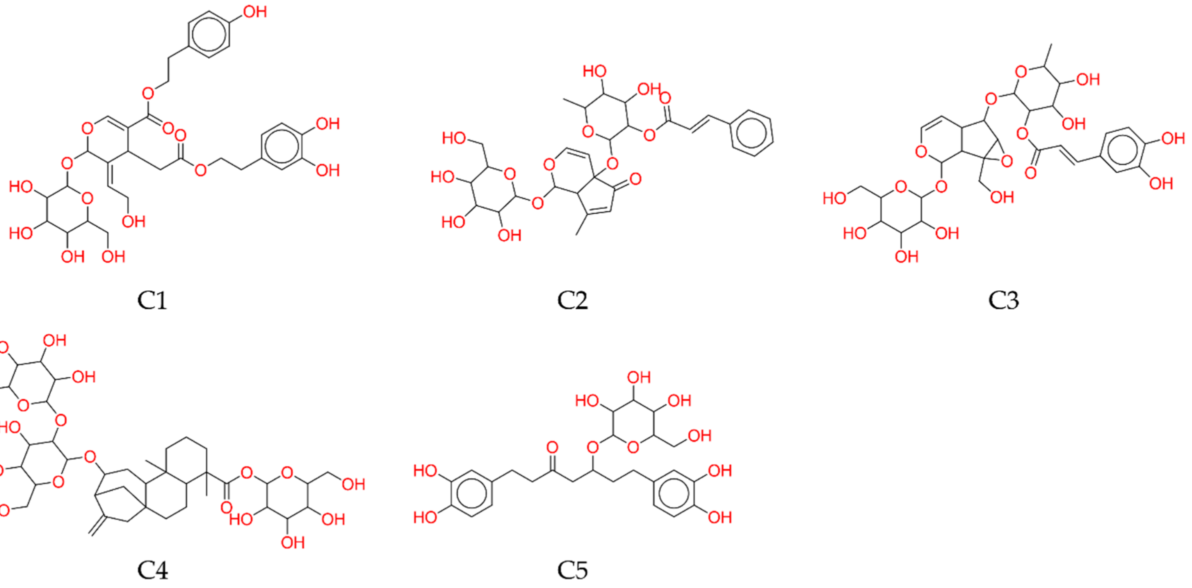

Figure S5. Chemical structures of potential inhibitors targeting nsp10–nsp16 interface.

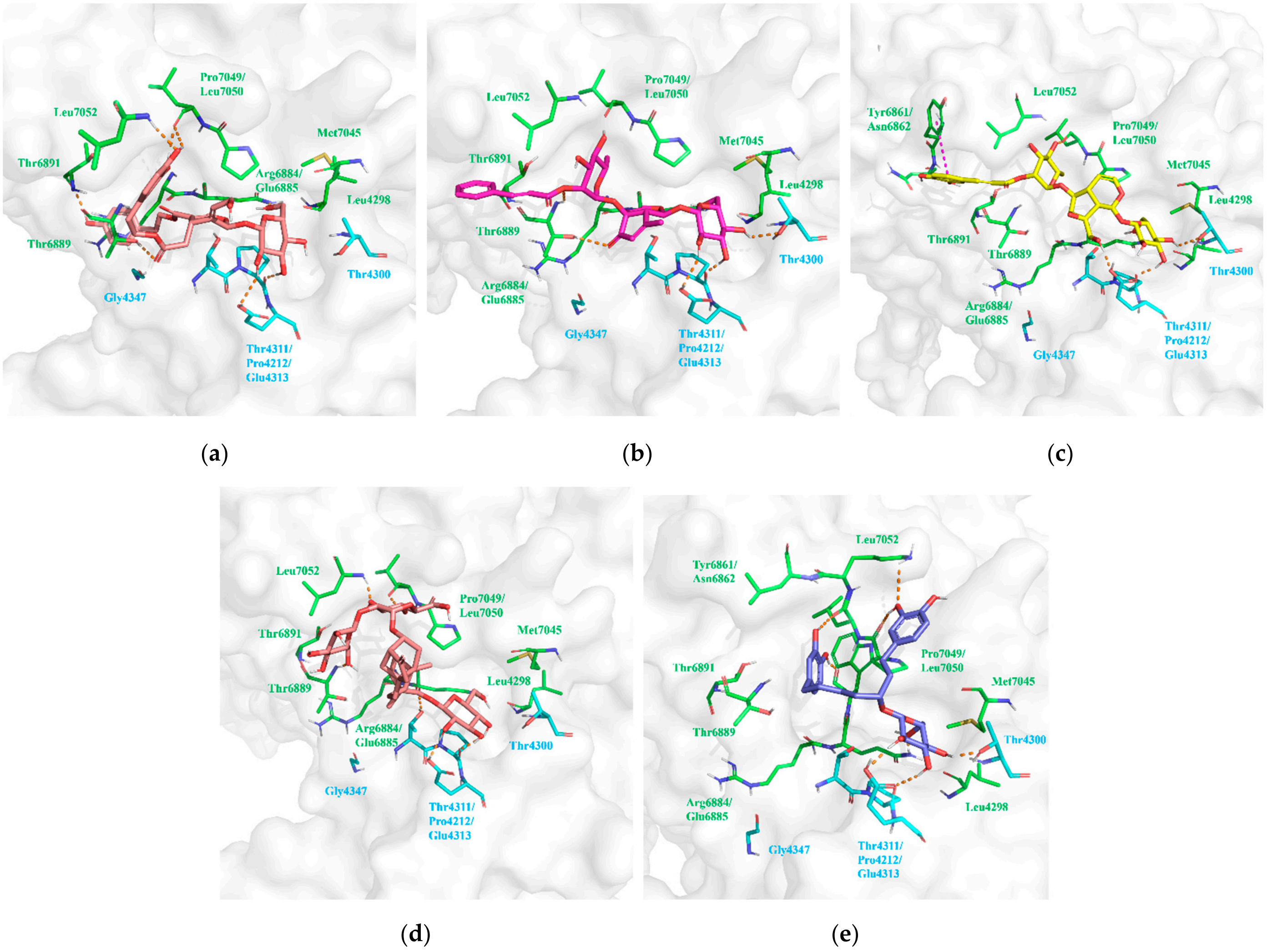

Figure S6. A three–dimensional view of the binding interactions of C1–C5 (**a**-**e**) in the nsp10-nsp16 interface. Amino acids of the nsp16 and nsp10 are displayed by green and cyan sticks respectively, and hydrogen bonds are displayed by orange dashed lines.

Table S4. Molecular docking results of potential nsp10–nsp16 interface inhibitors.

Code	Compound	Molecular Weight	LogP	Docking Score (kcal/mol)	H-Bond Interaction	π-π Stacking Interaction
C1	ZINC67911283	662.6	-0.10	-11.24	Gln6885, Thr6889, Thr6891, Leu7050(2) [1], Leu7052, Pro4312, Glu4313	\
C2	ZINC67912643	620.6	-0.76	-10.39	Gln6885, Thr6889, Leu7050, Thr4300, Pro4312, Glu4313	\
C3	ZINC95785585	670.6	-2.36	-9.98	Asn6862, Gln6885, Leu7050, Thr4300, Pro4312, Glu4313	Tyr6861
C4	ZINC253387786	804.9	-1.10	-9.82	Gln6885, Thr6889, Leu7050, Leu7052, Thy4311, Pro4312, Glu4313	\
C5	ZINC72320248	508.5	0.45	-9.35	Gln6885, Trp6886, Pro7049, Leu7050, Lys7051, Thr4300, Pro4312, Glu4313	\

[1] This represents the compound forms two hydrogen bonds with the same amino acid.

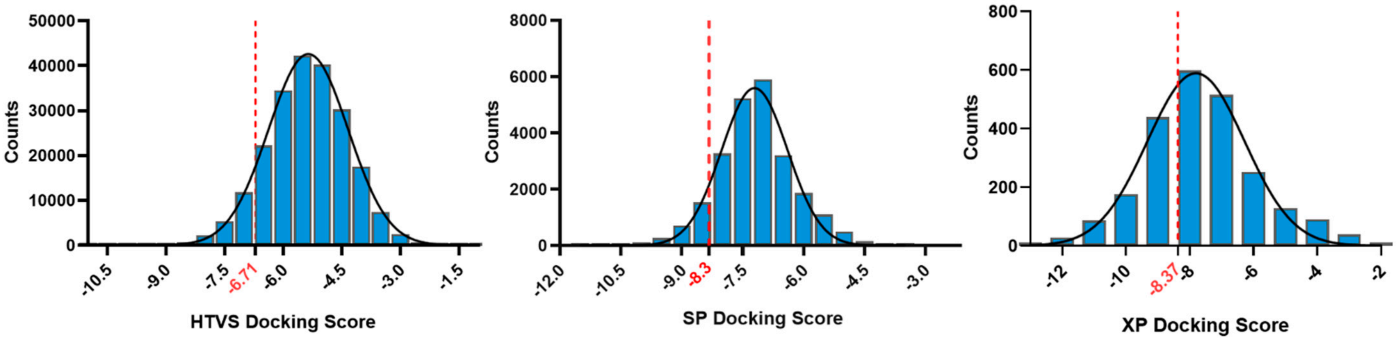

(**a**)

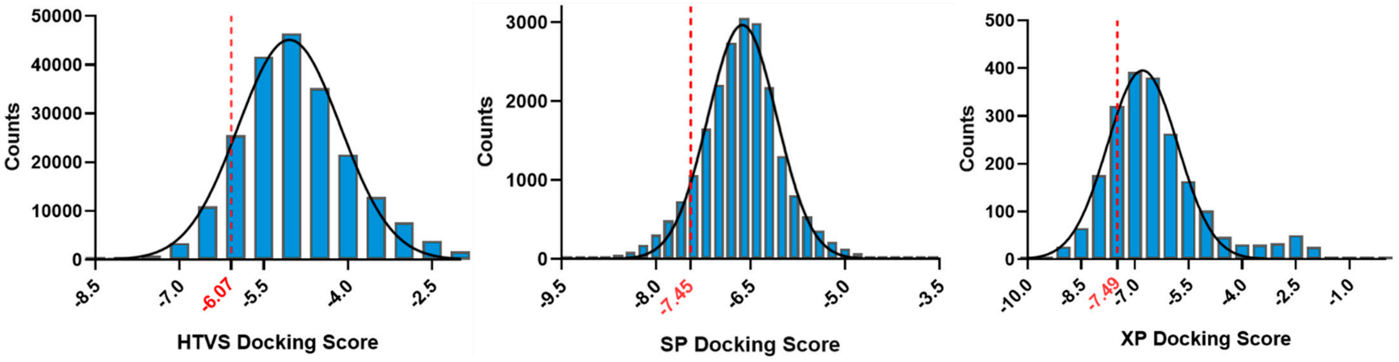

(**b**)

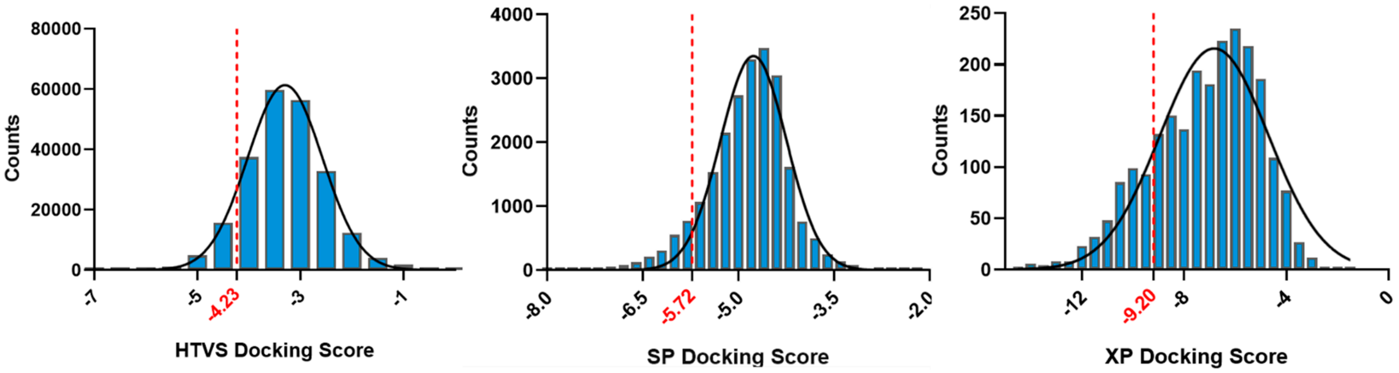

(**c**)

Figure S7. Docking score distribution of glide HTVS, SP and XP docking steps in VS of compounds targeting the SAM binding sites of nsp14 (**a**) and nsp16 (**b**) and the nsp10–nsp16 interface (**c**). The docking score at the top 10% position was employed as the cut–off threshold (red dashed lines) at the HTVS and SP screening steps, and the docking score at the top 20% position was used as cut–off threshold in the Glide XP screening step.

Table S5. Predicted ADMET properties of potential inhibitors targeting SAM binding site of SARS-Cov-2 nsp14 by pkCSM.

Property	Model Name	A1	A2	A3	A4	A5	A6	A7	A8	A9
absorption	water solubility	-3.421	-2.914	-4.192	-2.892	0.794	-3.528	-2.951	-3.097	-3.103
	intestinal absorption (human)	89.856	58.843	91.591	77.931	71.035	93.637	66.031	88.526	69.316
distribution	VDss (human)	-0.104	-0.102	-0.174	-0.054	0.016	-0.016	-0.351	0.462	0.667
metabolism	CYP2D6 substrate	No	No	No	No	No	No	No	No	No
	CYP3A4 substrate	Yes	No	Yes	No	No	Yes	No	No	No
	CYP1A2 inhibitor	Yes	No	No	No	No	Yes	No	Yes	No
	CYP2C19 inhibitor	Yes	No	Yes	No	No	Yes	No	No	No
	CYP2C9 inhibitor	Yes	No	Yes	No	No	No	No	No	No
	CYP2D6 inhibitor	No	No	No	No	No	No	No	No	No
	CYP3A4 inhibitor	No	No	Yes	No	No	Yes	No	No	No
excretion	total Clearance	-0.071	0.766	0.015	0.807	0.683	0.1290	0.794	0.589	0.62
	renal OCT2 substrate	No	No	No	No	No	Yes	No	No	No
toxicity	ames toxicity	No	No	No	Yes	No	No	Yes	No	No
	hERG I inhibitor	No	No	No	No	No	No	No	No	No
	hERG II inhibitor	Yes	No	No	No	No	Yes	Yes	Yes	Yes
	hepatotoxicity	Yes	No	Yes	Yes	Yes	Yes	No	Yes	Yes
	skin sensitisation	No	No	No	No	No	No	No	No	No

Table S6. Predicted ADMET properties of potential inhibitors targeting SAM binding site of SARS-Cov-2 nsp16 by pkCSM.

Property	Model Name	B1	B2	B3	B4	B5	B6	B7	B8
absorption	water solubility	-3.135	-3.318	-4.541	-3.51	-5.207	-4.29	-4.39	-3.758
	intestinal absorption (human)	94.325	93.459	96.145	87.708	93.317	95.45	93.473	91.947
distribution	VDss (human)	1.609	0.856	1.643	0.837	-0.291	1.03	1.102	1.798
metabolism	CYP2D6 substrate	Yes	No	Yes	Yes	No	Yes	No	No
	CYP3A4 substrate	Yes	No	Yes	Yes	Yes	Yes	Yes	Yes
	CYP1A2 inhibitor	No	No	Yes	No	Yes	Yes	Yes	Yes
	CYP2C19 inhibitor	No	No	Yes	Yes	Yes	No	No	No
	CYP2C9 inhibitor	No	No	No	No	Yes	No	No	No
	CYP2D6 inhibitor	Yes	No	Yes	Yes	No	Yes	Yes	Yes
	CYP3A4 inhibitor	No	No	Yes	Yes	Yes	Yes	No	No
excretion	total Clearance	1.163	0.634	1.22	0.711	0.206	1.063	1.049	1.117
	renal OCT2 substrate	Yes	No	No	Yes	No	No	No	No
toxicity	ames toxicity	No	No	Yes	No	No	No	No	Yes
	hERG I inhibitor	No	No	No	Yes	No	No	No	No
	hERG II inhibitor	Yes	No	Yes	Yes	Yes	Yes	Yes	Yes
	hepatotoxicity	Yes	Yes	Yes	Yes	Yes	Yes	Yes	No
	skin sensitisation	No	No	No	No	No	No	No	No

Table S7. Predicted ADMET properties of potential SARS-Cov-2 nsp10–nsp16 interface inhibitors by pkCSM.

Property	Model Name	C1	C2	C3	C4	C5
absorption	water solubility	-2.97	-3.26	-2.56	-2.56	-3.07
	intestinal absorption (human)	29.41	18.79	12.42	0	26.85
distribution	VDss (human)	0.68	0.24	0.38	-0.52	0.12
metabolism	CYP2D6 substrate	No	No	No	No	No
	CYP3A4 substrate	No	Yes	No	No	No
	CYP1A2 inhibitor	No	No	No	No	No
	CYP2C19 inhibitor	No	No	No	No	No
	CYP2C9 inhibitor	No	No	No	No	No
	CYP2D6 inhibitor	No	No	No	No	No
	CYP3A4 inhibitor	No	No	No	No	No
excretion	total Clearance	0.69	1.02	0.70	0.75	0.71
	renal OCT2 substrate	No	No	No	No	No
toxicity	ames toxicity	No	No	No	No	No
	hERG I inhibitor	No	No	No	No	No
	hERG II inhibitor	No	Yes	Yes	Yes	No
	hepatotoxicity	No	No	No	No	No
	skin sensitisation	No	No	No	No	No

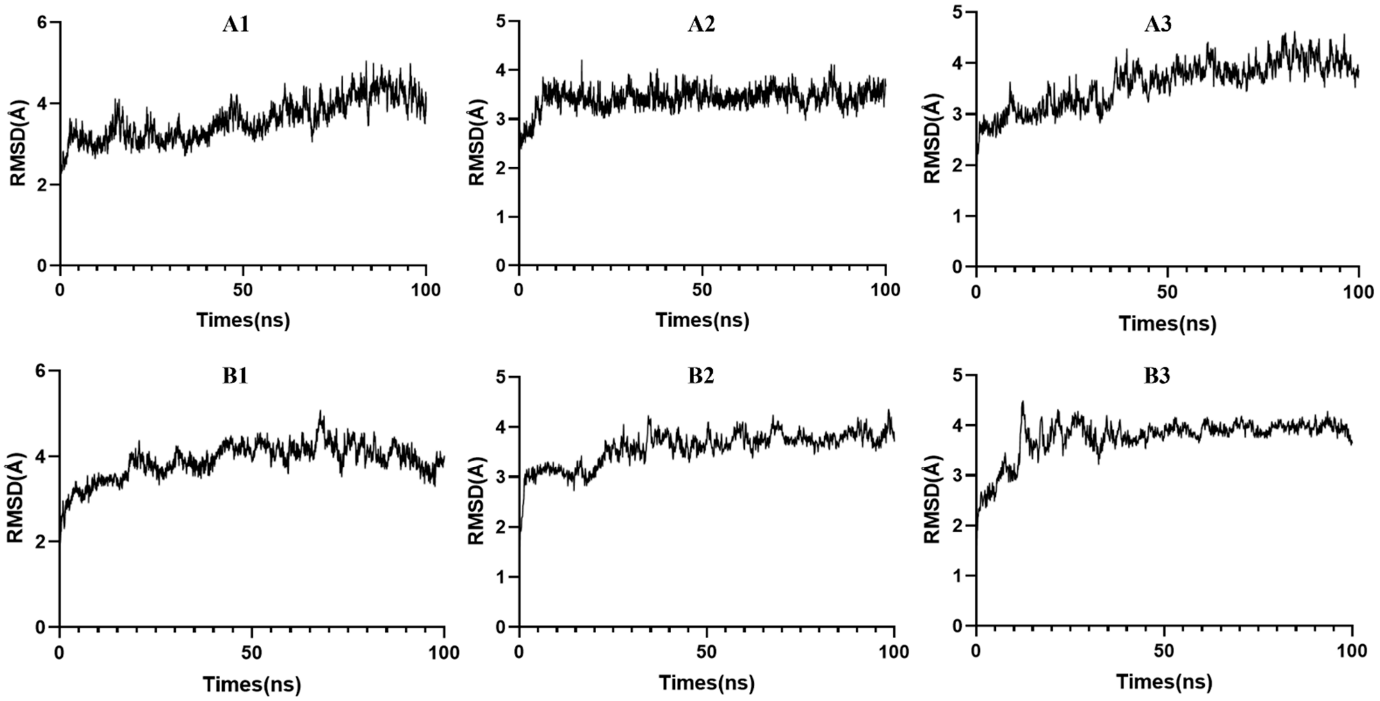

(**a**)

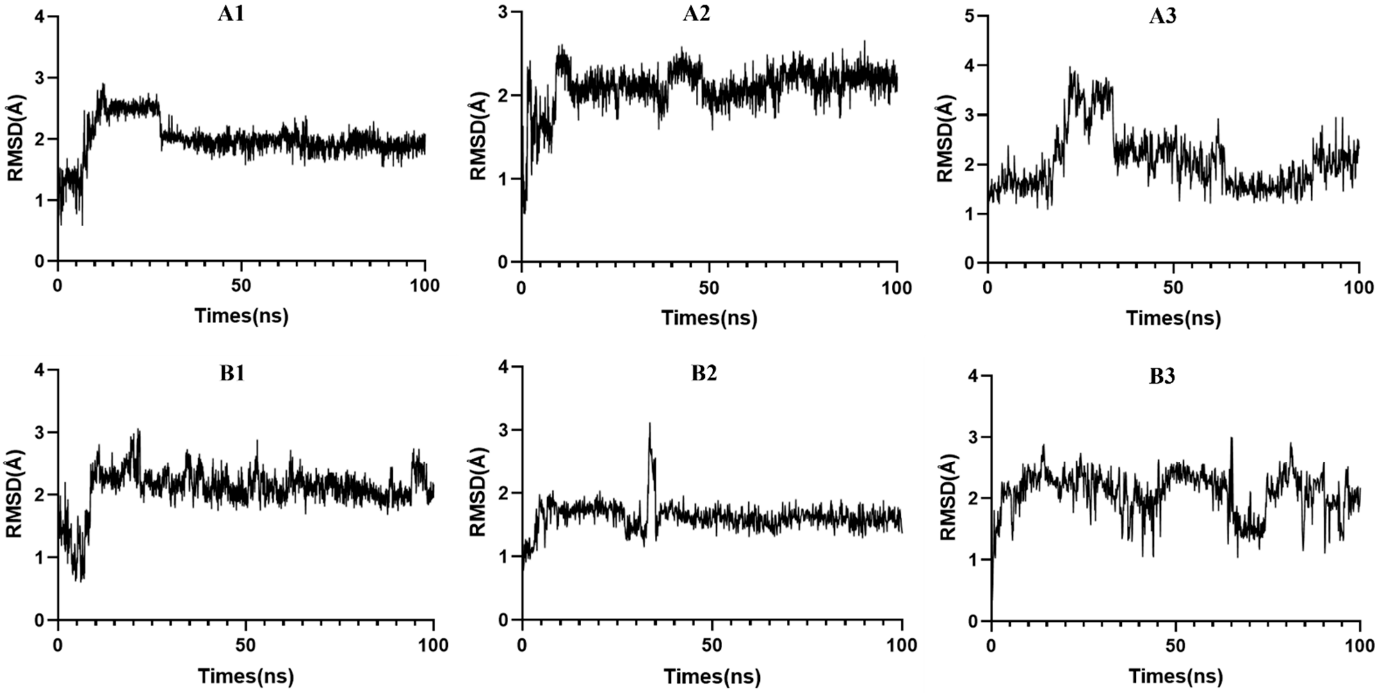

(**b**)

Figure S8. The RMSD of protein backbone (a) and ligands (b) as a function of time for the 6 hits bound to SARS-CoV-2 nsp14/nsp16 SAM binding site in the 100 ns MD simulations.

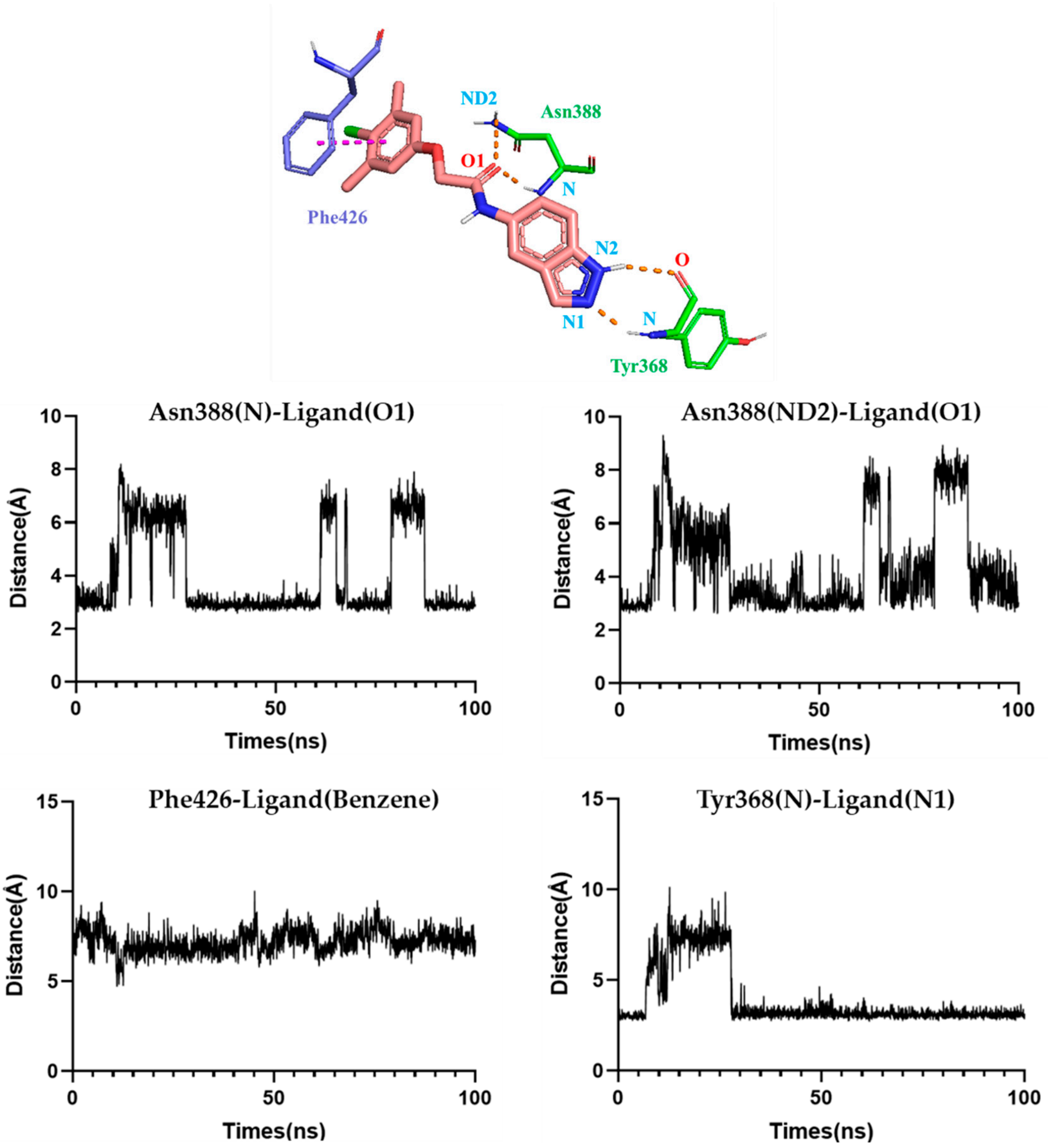

Figure S9. The predicted 3D binding mode of compound A1 bound to SARS-CoV-2 nsp14, and the distances that describe the binding interactions between the ligand and protein as a function of time in the 100 ns MD simulation. Amino acids in the SAM–adenine and RNA cap binding cavities are displayed by green and purple sticks respectively. The hydrogen bonds are displayed by orange dashed lines, and π-π stacking interactions are displayed by pink dashed lines.

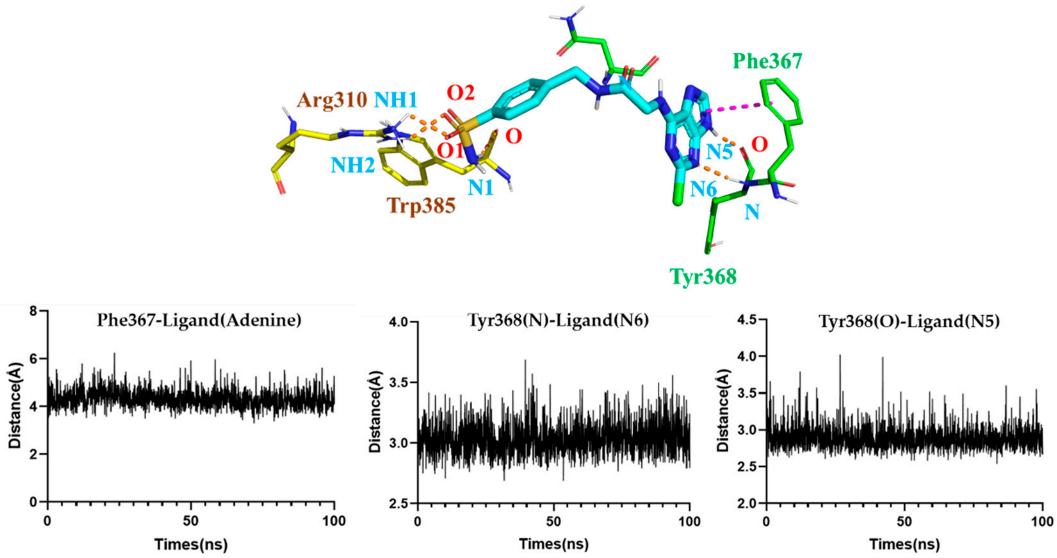

Figure S10. The predicted 3D binding mode of compound A2 bound to SARS-CoV-2 nsp14, and the distances that describe the binding interactions between the ligand and protein as a function of time in the 100 ns MD simulation. Amino acids in the SAM–adenine and SAM–tail cavities are displayed by green and yellow sticks respectively. The hydrogen bonds are displayed by orange dashed lines, and π-π stacking interactions are displayed by pink dashed lines.

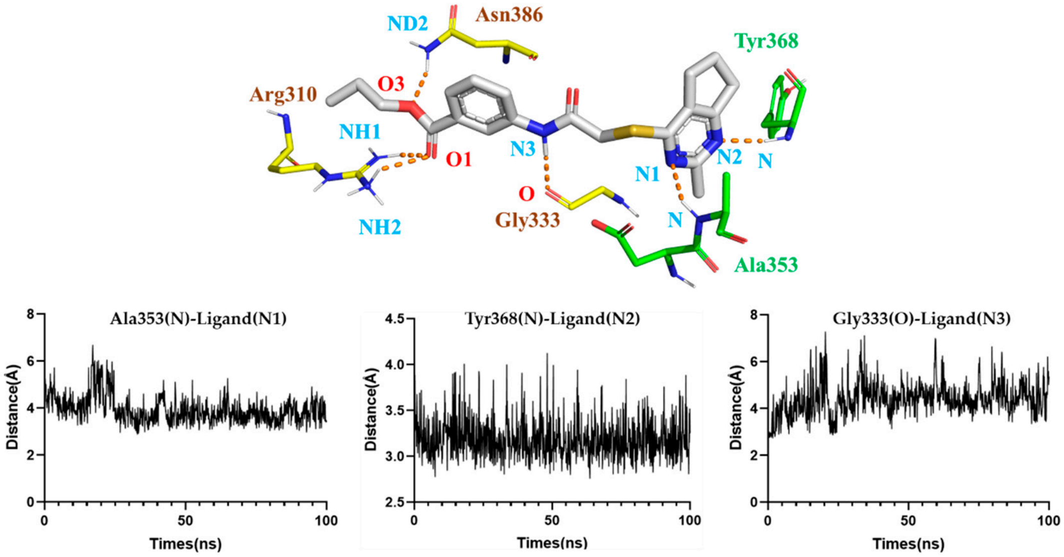

Figure S11. The predicted 3D binding mode of compound A3 bound to SARS-CoV-2 nsp14, and the distances that describe the binding interactions between the ligand and protein as a function of time in the 100 ns MD simulation. Amino acids in the SAM–adenine and SAM–tail cavities are displayed by green and yellow sticks respectively. The hydrogen bonds are displayed by orange dashed lines.

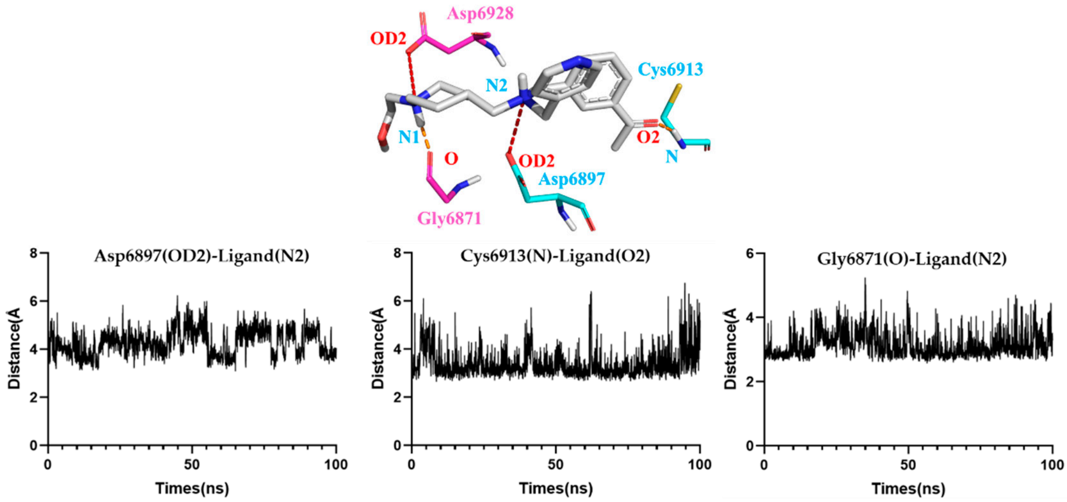

Figure S12. The predicted 3D binding mode of compound B1 bound to SARS-CoV-2 nsp16, and the distances that describe the binding interactions between the ligand and protein as a function of time in the 100 ns MD simulation. Amino acids in the SAM–adenine and SAM–tail cavities are displayed by cyan and pink sticks respectively. The hydrogen bonds are displayed by orange dashed lines, and salt bridge interactions are displayed by red dashed lines.

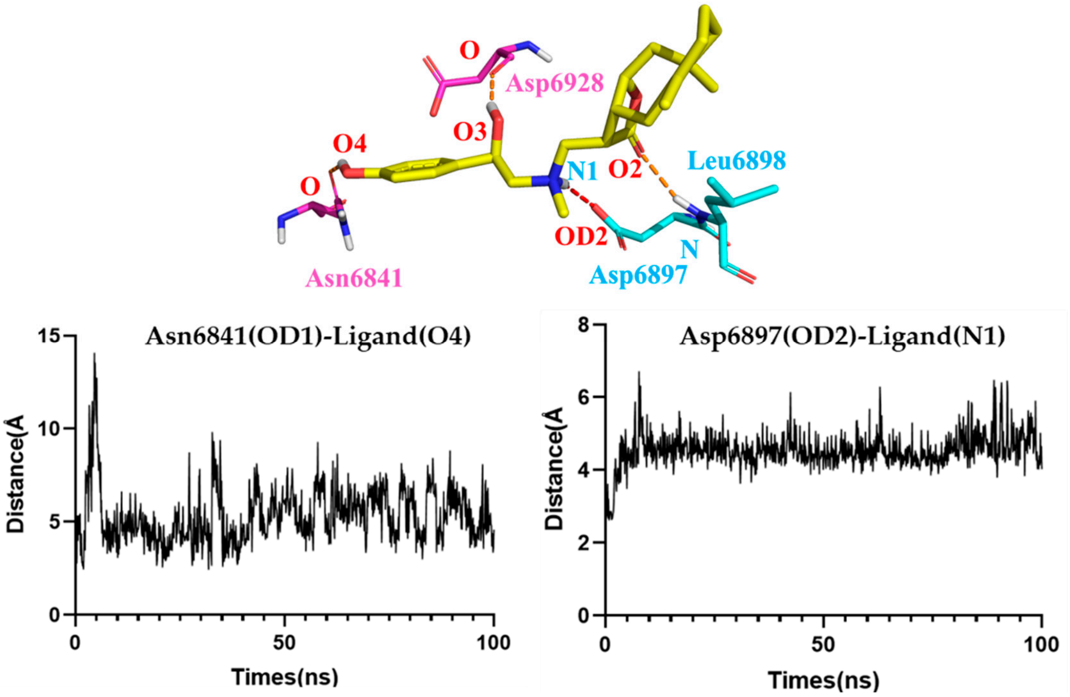

Figure S13. The predicted 3D binding mode of compound B2 bound to SARS-CoV-2 nsp16, and the distances that describe the binding interactions between the ligand and protein as a function of time in the 100 ns MD simulation. Amino acids in the SAM–adenine and SAM–tail cavities are displayed by cyan and pink sticks respectively. The hydrogen bonds are displayed by orange dashed lines and salt bridge interactions are displayed by red dashed lines.

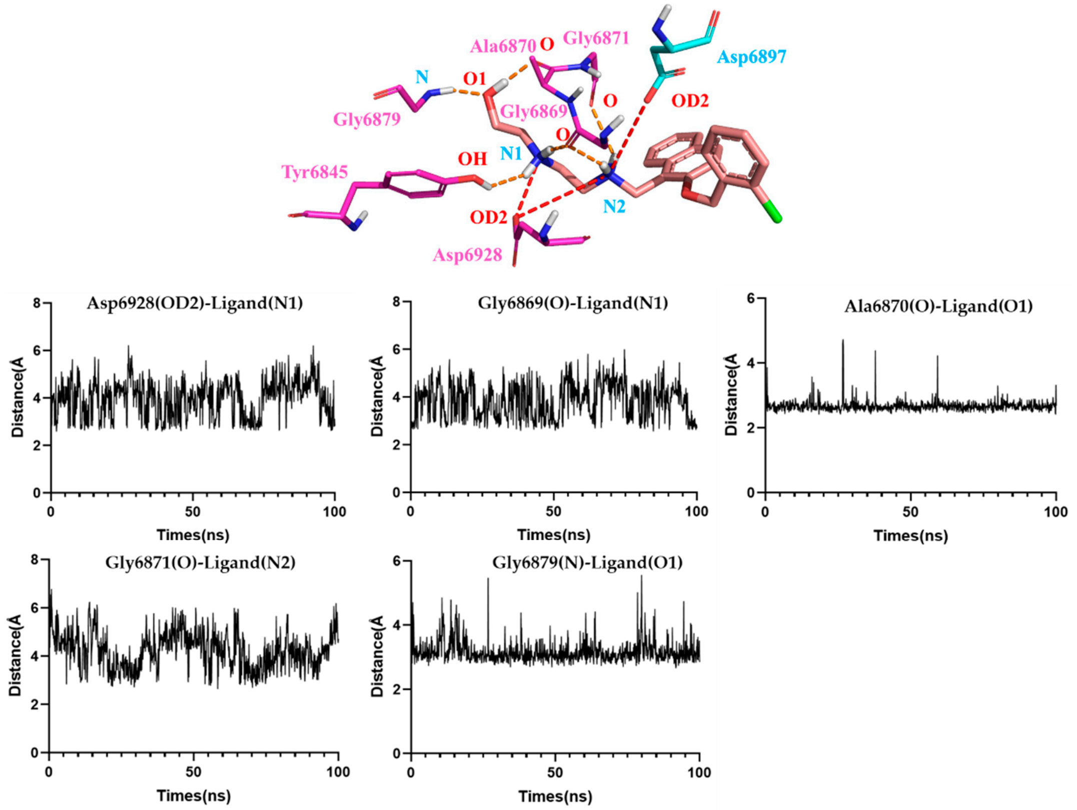

Figure S14. The predicted 3D binding mode of compound B3 bound to SARS-CoV-2 nsp16, and the distances that describe the binding interactions between the ligand and protein as a function of time in the 100 ns MD simulation. Amino acids in the SAM–adenine and SAM–tail cavities are displayed by cyan and pink sticks respectively. The hydrogen bonds are displayed by orange dashed lines, and salt bridge interactions are displayed by red dashed lines.
